# Diels–Alder Adducts from *Maytenus chiapensis*

**DOI:** 10.3390/ijms27073318

**Published:** 2026-04-07

**Authors:** Ulises G. Castillo, Morena L. Martínez, Marvin J. Núñez, Aday González-Bakker, José M. Padrón, Nathália Nocchi, Eduardo Hernández-Álvarez, Ignacio A. Jiménez, Isabel L. Bazzocchi

**Affiliations:** 1 Laboratorio de Investigación en Productos Naturales, Facultad de Química y Farmacia, Universidad de El Salvador, San Salvador 1101, El Salvador; ulises.guardado@ues.edu.sv (U.G.C.); morena.martinez@ues.edu.sv (M.L.M.); marvin.nunez@ues.edu.sv (M.J.N.); 2 Instituto Universitario de Bio-Orgánica Antonio González, Departamento de Química Orgánica, Universidad de La Laguna, Av. Astrofísico Francisco Sánchez 2, 38206 La Laguna, Spain; alu0100947311@ull.edu.es (E.H.-Á.); ignadiaz@ull.edu.es (I.A.J.); 3 Instituto Universitario de Bio-Orgánica Antonio González, Universidad de La Laguna, Av. Astrofísico Francisco Sánchez 2, 38206 La Laguna, Spain; agonzaba@ull.edu.es (A.G.-B.); jmpadron@ull.edu.es (J.M.P.); nathalianocchi@ull.edu.es (N.N.); 4 Biotecnología Marina, Instituto Universitario de Bio-Orgánica Antonio González, Universidad de La Laguna, Unidad Asociada al IPNA-CSIC, 38206 La Laguna, Spain

**Keywords:** Celastraceae, *Maytenus chiapensis*, hetero-Diels-Alder adducts, cancer, acetylcholinesterase

## Abstract

Natural products from plants have played an important role in cancer and neurodegenerative diseases. In this context, the root bark of *Maytenus chiapensis* (Celastraceae) was investigated to examine its chemical constituents and potential biological activities. Chromatographic separation of the root bark extract yielded a new Diels–Alder adduct (morenine) formed by a triterpenophenolic moiety derived from tingenone and a bicyclic guaiane-type sesquiterpene linked through a 1,4-dioxane bridge. In addition, eight previously reported Diels–Alder adducts—retusonine and cheiloclines A–D and F–H—were isolated, together with their biosynthetic precursors, the quinone-methide triterpenoids (QMTs) pristimerin and tingenone. Structural elucidation was achieved through detailed 1D and 2D NMR spectroscopic analyses. The adducts were tested for cytotoxicity against six cancer cell lines (A549, SW1573, MIA PaCa-2, T-47D, HeLa, and WiDr cell lines), showing moderate-to-low activity compared with their precursors. Continuous live cell imaging identified apoptosis and vacuole formation as the main modes of action of pristimerin in SW1573 cells. Moreover, acetylcholinesterase inhibition assays revealed that cheiloclines B–D, F, and H exhibited up to 50% inhibition. These findings reinforce the potential of Celastraceae species as a source of unique and complex compounds and enhance our understanding of their therapeutic potential.

## 1. Introduction

Natural products and derivatives have been the main source of newly approved drugs by the FDA in recent decades [1,2]. Additionally, it has been reported that approximately 65% of the over 400,000 isolated and characterized natural compounds are of plant origin, while only 0.5% come from animals or marine organisms [3].

In the search for new drugs, the Celastraceae family has attracted attention due to its ability to produce secondary metabolites with complex structures and promising pharmacological activities [4,5,6,7,8,9]. This family includes woody plants such as trees, shrubs, and vines, distributed throughout tropical regions worldwide, with some species also present in temperate zones [10,11], comprising 96 genera and more than 1350 species [10]. These species are known to produce a wide range of secondary metabolites. Among these, macrocyclic alkaloids, quinone-methide triterpenoids (QMTs), and dihydro-β-agarofuran sesquiterpenes are particularly abundant, with the latter two serving as key chemotaxonomic markers of the family [11,12,13]. A distinctive feature of the Celastraceae species is the exclusive occurrence of Diels–Alder adducts (DAAs), a unique class of natural products. More than 80 DAAs had been reported as of 2019 from Celastraceae, with triterpene dimers being the most prevalent (~76%), largely isolated from the *Maytenus* genus, one of the most extensively studied genera due to its ethnopharmacological significance [14].

*Maytenus chiapensis* is a native species distributed in México (Chiapas), Guatemala and El Salvador, and it mainly grows in tropical humid biomes [15]. Although several metabolites with diverse skeletal types and noteworthy biological activities have been reported from *M. chiapensis*, including QMTs and related celastroloids [16,17], the Diels–Alder adducts present in the root bark remain unexplored.

Furthermore, most Diels–Alder adducts (DAAs) reported from Celastraceae species correspond to triterpene dimers arising from a cycloaddition [4+2] between quinone-methide triterpenoids (QMTs) [14]. In contrast, hetero-Diels–Alder adducts, incorporating a sesquiterpene linked through a 1,4-dioxane ring, remain exceedingly rare. In fact, only a few examples have been reported from *Cheiloclinium hippocratioides* [18].

In continuing efforts to explore structurally complex constituents from Celastraceae species, the root bark extract of *M. chiapensis* afforded one previously undescribed hetero-Diels–Alder adduct, named morenine (**1**), together with known octacyclic adducts (**2**–**9**) and their QMT precursors (**10** and **11**). Their structures were established by comprehensive 1D/2D NMR experiments and HRMS data analysis. Furthermore, compounds **1**, **2**, **8**, **10** and **11** were evaluated for their antiproliferative effects on six human tumor cell lines, and the most active one, pristimerin, was selected to gain deeper insight into their mode of action in SW1573 cells by continuous live cell imaging. Acetylcholinesterase (AChE) inhibitory activity was also assessed for all available compounds (**2–11**).

## 2. Results and Discussion

### 2.1. Isolation and Structural Elucidation

**Figure 1:**
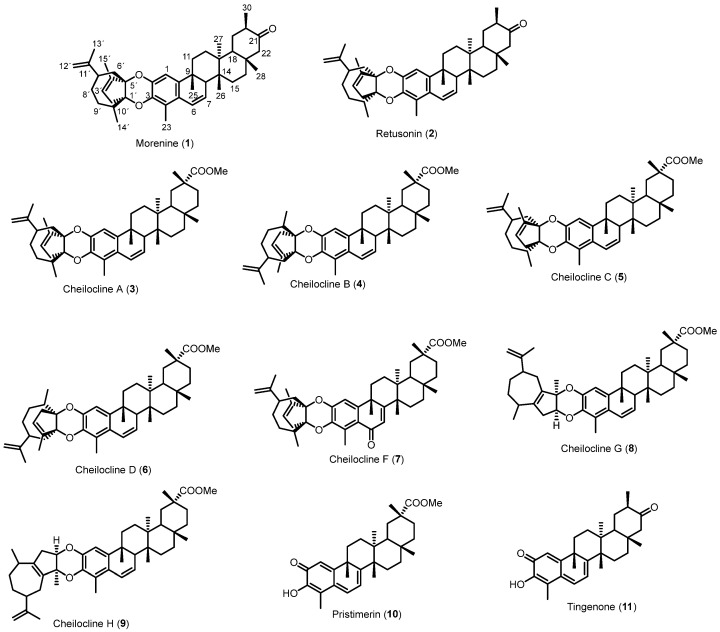
Chemical structures of compounds from *M. chiapensis* root bark.

The *n*-hexane–diethyl ether (1:1) extract of *M. chiapensis* root bark (27.2 g) was chromatographed over a silica gel column using increasing-polarity mixtures of *n*-hexane–dichloromethane, and subsequently dichloromethane–ethyl acetate, to afford 66 fractions that were combined according to TLC analysis, yielding eight subfractions (A to H). Fraction A was subjected to silica gel chromatography using mixtures of *n*-hexane/CH_2_Cl_2_ of increasing polarity as eluent and preparative TLC, yielding the Diels–Alder adducts (Figure 1) cheiloclines C (**5**) and G (**8**). Several chromatographic steps were done over fraction B to afford the adducts morenine (**1**), retusonine (**2**), and cheiloclines A (**3**), B (**4**), C (**5**), D (**6**), F (**7**), and H (**9**). Fractions D and E yielded the QMTs pristimerin (**10**) and tingenone (**11**) [18,19,20,21].

Compound **1** was isolated as a colorless lacquer and showed a specific rotation of ${\left[\alpha \right]}_{D}^{25}$ −34.5, and ESI-HRMS analysis revealed a pseudomolecular ion [M+Na]^+^ at *m*/*z* 645.4284 (Figure S1) consistent with a molecular formula of C_43_H_58_O_3_, indicating 15 degrees of unsaturation. The most representative signals in the ^1^H NMR spectrum (Table 1, Figure S1) were five singlet methyl groups at δ_H_ 2.17 (Me-23), 1.33 (Me-27), 1.10 (Me-26), 1.00 (Me-25), and 0.95 (Me-28); one doublet methyl at δ_H_ 1.01 (Me-30, *J* = 5.9 Hz); two vinylic protons as double doublets at δ_H_ 6.67 (H-6, *J* = 2.8, 9.8 Hz) and 5.89 (H-7, *J* = 2.8, 9.8 Hz); and one aromatic proton as a singlet at δ_H_ 6.56 (H-1). These data were confirmed by analysis of the ^13^C NMR spectrum (Table 1, Figure S1), showing, as key signals, a ketone at δ_C_ 214.8 (C-21) and a disubstituted double bond at δ_C_ 128.2 (C-7) and 110.5 (C-6), as well as signals consistent with a penta-substituted aromatic ring featuring an *ortho*-catechol system, with characteristic resonances at δ_C_ 143.4 (C-3), 142.1 (C-10), 141.9 (C-2), 125.1 (C-5), 120.4 (C-4), and 102.6 (C-1). Moreover, the complete ^1^H and ^13^C NMR assignments were established based on 2D NMR experiments (edited HSQC and HMBC, Figure S1), which allowed unambiguous correlation of each proton with its corresponding carbon and key long-range connectivity. Comparison of these data with the literature allowed us to propose a *nor*-triterpenophenol unit corresponding to a derivative of 9,11-dihydroisotingenone III [22].

Moreover, the ^1^H NMR spectrum exhibited the following key signals: a vinylic proton at δ_H_ 5.40 (H-3′, s); two vinylic protons of the isopropylidene group at δ_H_ 4.78 (1H, s, H-12′a) and 4.72 (1H, s, H-12′b); and a methyl singlet at δ_H_ 1.78 (Me-13′). Two additional methyl signals were observed, one singlet at δ_H_ 2.01 (Me-15′) and one doublet at δ_H_ 1.15 (Me-14′, *J* = 7.22 Hz). These assignments were supported by the ^13^C NMR spectrum, which revealed a trisubstituted double bond with resonances at δ_C_ 142.2 (C-4′) and 125.0 (C-3′), as well as two methyl signals at δ_C_ 20.5 (Me-14′) and 11.5 (Me-15′). Additionally, an isopropylidene system was identified with signals at δ_C_ 151.9 (C-11′), 108.8 (C-12′), and 20.9 (Me-13′), along with two oxygenated quaternary carbons at δ_C_ 97.1 (C-5′) and 91.2 (C-1′), indicating that a sesquiterpene moiety linked to the *nor*-triterpenophenol unit corresponds to a bicyclic guaiane-type sesquiterpene, specifically derived from guaia-1(5),3(4),11(3)-triene [23]. These assignments for the sesquiterpene unit were likewise confirmed by HSQC and HMBC correlations, which supported the presence of the trisubstituted double bond and the isopropylidene system, as summarized in Table 1.

**Figure 2:**
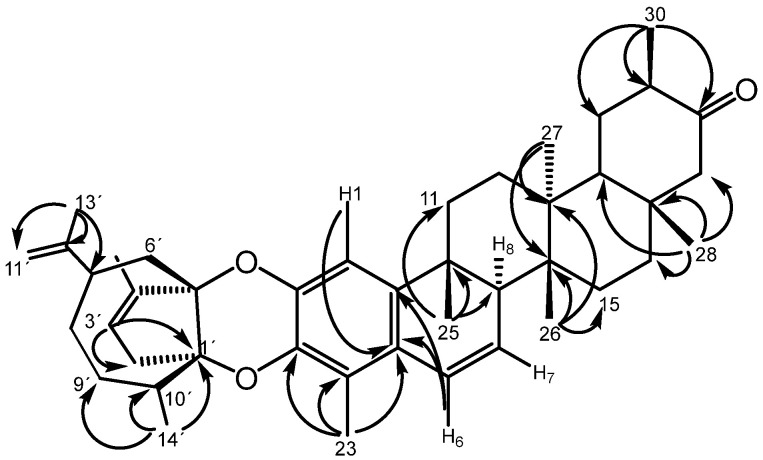
Selected HMBC (^1^H-^13^C) long-range correlations for morenine (**1**).

The triterpenoid unit is characterized as pentacyclic, possessing one aromatic ring, a double bond in ring B, and a ketone group located in ring E. The sesquiterpene unit presents four degrees of unsaturation, two attributable to double bonds between C-3′/C-4′ and C-11′/C-12′, and two corresponding to ring structures. The multiple correlations observed in the HMBC experiment (Heteronuclear Multiple-Bond Correlation) of signals at δ_C_ 97.1 (C-5′) and 91.2 (C-1′) suggest a bridged bicyclic system, with these quaternary carbons being in bridgehead positions (Table 1, Figure 2 and Figure S1). The three-bond correlations observed of the protons of Me-14′ with the methylene carbon C-9′ and the quaternary carbon C-1′ enabled the elucidation of a 1,4-dioxane system in the compound. In this system, the quaternary carbons C-1′ and C-5′ of the sesquiterpene unit serve as bridgehead atoms.

**Figure 3:**
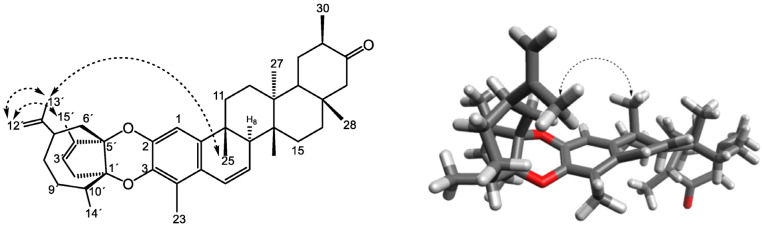
Key NOE (Nuclear Overhauser Effect) correlations for morenine (**1**). Dashed arrows indicate selected NOE correlations. The 3D model illustrates the spatial arrangement supporting the relative stereochemistry.

To determine the relative stereochemistry, a 2D homonuclear ^1^H–^1^H ROESY experiment was carried out (Figure S1 in Supplementary Materials). The most diagnostic and reliable correlation was observed between protons Me-13′ and Me-25, suggesting a β-orientation of the cycloheptane ring within the sesquiterpene moiety. Additionally, this experiment enabled the determination of the regio-substitution pattern of the 1,4-dioxane system, confirming linkages at [2-*O*-5′] and [3-*O*-1′] (Figure 3). A 2D homonuclear ^1^H–^1^H COSY experiment was also performed, which established the *spin* systems between H-6/H-7/H-8 and H-3′/H-2, defining the main *spin* systems of the triterpenoid and sesquiterpene units, respectively, thus providing supporting evidence for the connectivity pattern (Figure S1).

In this previously unreported compound, the formation of the 1,4-dioxane ring may follow a mechanism analogous to that proposed for the formation of triterpene dimers [13,18], in which the triterpenophenol undergoes oxidation at ring A to an *ortho*-quinone and this acts as the diene, while the conjugated system of the guaiane-derived moiety (C-1′ and C-5′) behaves as the dienophile through a hetero-Diels–Alder reaction.

Morenine (**1**) represents a structurally novel hetero-Diels–Alder adduct, distinguished from previously reported congeners by its unique combination of a *nor*-triterpenophenol (derived from 9,11-dihydroisotingenone) and a bicyclic guaiane sesquiterpene linked via a 1,4-dioxane bridge [18]. Unlike the predominant triterpene dimers (DAAs) in the Celastraceae, which are homo-Diels–Alder adducts, morenine exemplifies a hetero-Diels–Alder pathway involving an *ortho*-quinone diene and a conjugated dienophile from the guaiane scaffold. This finding not only enriches the structural diversity of DAAs but also supports their chemotaxonomic significance in this botanical family. DAAs and QMTs consistently co-occur and may act as lineage-specific markers.

The structures of compounds **2**–**11** were confirmed by comparison of their ^1^H and ^13^C NMR data with those reported in the literature. The respective spectroscopic data and references are compiled in the Supplementary Materials (Figures S2–S11, Table S1). Overall, the excellent agreement between their NMR data and the published values confirmed that these metabolites correspond to previously reported natural products [18,21,24].

### 2.2. Biological Activities

#### 2.2.1. Antiproliferative Activity on Cancer Cell Lines

Morenine (**1**), the previously reported adducts (**2**–**9**) and the QMT precursor units (**10** and **11**) were evaluated for their antiproliferative activity against six tumor cell lines: A549, SW1573, MIA PaCa-2, T-47D, HeLa, and WiDr. Cheiloclines A (**3**), C (**5**), D (**6**), and F (**7**) could not be evaluated owing to limited solubility under experimental conditions. The most relevant findings are shown in Table 2.

Based on the 50% growth inhibition (GI_50_) values, QMTs exhibited the highest activity among the compounds evaluated, with pristimerin (**10**) being most active compared to the two positive controls used against all the evaluated cell lines, results that are consistent with previous reports [6,22,25,26,27]. Moreover, the cytotoxicity of this QM on SW1573 (non-small-cell lung) (GI_50_ 1 µM) is reported herein for the first time.

In addition, morenine (**1**), retusonine (**2**), and cheilocline G (**8**) also exhibited activity, although to a lesser extent. The most active adduct was cheilocline G (**8**), with GI_50_ values ranging from 26.7 to 71 µM. The A549 cell line (non-small-cell human lung carcinoma) was the most sensitive.

The triterpenoid quinone methides tingenone (GI_50_ values between 1.5 and 16.4 µM) and pristimerin (GI_50_ values between 0.15 and 0.9 µM) displayed higher activity than their corresponding cycloadducts (GI_50_ values 27–89 µM), highlighting the remarkable role of the functional group on ring E. Moreover, sesquiterpene–tingenone adducts, morenine (GI_50_ values 78–91 µM) and retusonine (GI_50_ values 44–85 µM), exhibited higher activity than their sesquiterpene–pristimerin congeners, as evidenced by the antiproliferative activity of compounds **1** and **2** relative to **4** and **9**. In addition, within the same series, compound **9** was inactive (GI_50_ > 100 µM), whereas adduct **8** exhibited significant activity (GI_50_ values between 26.7 and 71 µM). Therefore, the nature of the cycloadduct and its regioisomeric arrangement played a crucial role in the observed activity.

Although the cytotoxic activity of the isolated adducts is lower compared to their precursor triterpenoid units, pristimerin (**10**) and tingenone (**11**), the evaluation of their therapeutic potential should not be based solely on potency. These precursor compounds are known to interact with a wide array of biological targets and exhibit a broad spectrum of biological activities [7,12,25,26], while contributing to their cytotoxic efficacy, also leading to undesirable off-target effects that have hindered their development as drugs [28]. In contrast, DAAs, due to their higher molecular weight and increased structural complexity, appear to engage in fewer interactions with biological targets, as reported by Bazzocchi et al. [14]. This reduced promiscuity may confer greater selectivity and a more favorable safety profile. Consequently, despite their moderate cytotoxicity, these adducts represent promising candidates for further investigation, particularly through toxicity assays in normal cell lines or whole-organism models to better understand their therapeutic index and selectivity.

#### 2.2.2. Getting Insights on SW1573 Cell Death by Pristimerin

Pristimerin is used in traditional Chinese medicine to treat various cancers, and recent studies have identified alterations in cellular events and molecular signaling targets under pristimerin treatment. Molecular factors and pathways are associated with its anticancer activities, including induction of cell cycle arrest, apoptosis, and autophagy [29].

**Figure 4:**
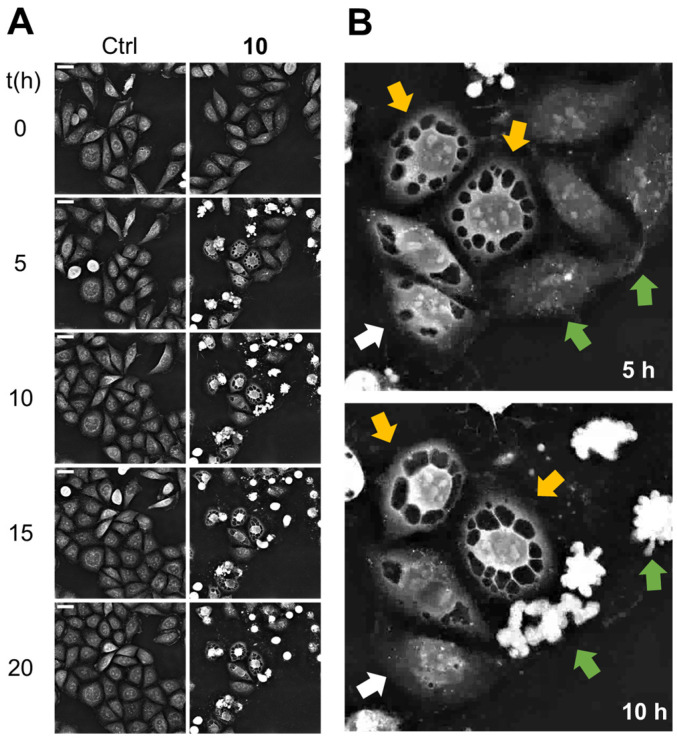
(**A**): Representative snapshots of SW1573 untreated cells (Ctrl) and cells exposed to pristimerin (**10**) (1 µM, 20 h). Scale bar: 30 µM. (**B**): Detailed view of the phenotypic changes induced by pristimerin (**10**). Yellow arrows indicate vacuolated cells. Green arrows indicate the presence of apoptotic cells. The white arrow points to a vacuolated cell that recovered from the effects.

In the present study, this triterpenoid was evaluated for comparative purposes with the isolated adducts, resulting in being the most active compound against all the assayed cell lines. Despite pristimerin having been assayed on numerous cell lines, to our knowledge, this is the first report of its effect on a non-small-cell lung carcinoma (SW1573) cell line. Therefore, we decided to conduct an analysis through continuous live cell imaging (Figure 4 and Supplementary Video S1) to observe its predominant mode of action as well as to analyze the potency, in addition to cell death kinetics [30].

For this study, SW1573 cells were exposed to pristimerin (**10**), at a dose of 1 μM (4 × GI_50_) for 20 h. The recording took place at intervals of 5 min. Figure 4 shows representative snapshots taken at different time points. The exposure of SW1573 cells to pristimerin (**10**) resulted in two distinct outcomes (Figure 4B). Firstly, a population of cells underwent apoptosis, characterized by classical hallmarks such as cell shrinkage, nuclear condensation, and subsequent fragmentation. Simultaneously, multiple large vesicles accumulated in the cytoplasm of other cells. Both effects manifested within 2 h of exposure, underscoring the rapid action of pristimerin (**10**). Interestingly, a single cell that initially exhibited vacuolization (Figure 4B, white arrow) reversed this phenotype, with the vesicles being completely reabsorbed after 10 h of exposure. Vacuolization is a recognized mechanism of drug resistance induced by pharmacological stress. Consequently, the reversal of this phenotype suggests a potential compensatory mechanism emerging at the early stages of treatment. This adaptative response might limit the overall pro-apoptotic efficacy of the treatment, suggesting that the initial cellular stress does not always lead to irreversible cell death. To the best of our knowledge, this is the first evidence of a potential resistance mechanism in cancer cells against pristimerin (**10**), indicating a possible adaptive response that should be confirmed in further studies.

#### 2.2.3. In Vitro Acetylcholinesterase Inhibitory Assay

Acetylcholinesterase (AChE) is an enzyme involved in the hydrolysis of the neurotransmitter acetylcholine and regulation of cholinergic signaling, whose inhibition may be therapeutically relevant in conditions associated with impaired cholinergic transmission, such as neurodegenerative disorders and neuromuscular dysfunctions [31].

Therefore, in the search for new AChE-targeting agents, the AChE inhibitory activity of the eight known DAAs (**2**–**9**) and their QMT precursors (**10** and **11**) was evaluated through in vitro screening by spectrophotometric Ellman’s method [32] with minor modification [33], using AChE from *Electrophorus electricus*. Rivastigmine was used as the reference standard. The AChE inhibitory activity of the tested compounds at a concentration of 100 µM was expressed as percentage inhibition values, which are summarized in Table 3. Morenine (**1**) could not be evaluated for AChE inhibition due to insufficient material remaining after exhaustive structural elucidation and cytotoxicity assays.

Based on the percentage inhibition values, the QMTs exhibited the highest activity among the evaluated compounds, consistent with the results observed in the cytotoxicity assays. The most active compounds were pristimerin (**10**) (64.3%), followed by tingenone (**11**) (50.9%), the triterpenoid precursor units of the adducts. These findings agree with previous reports indicating that structurally related pentacyclic triterpenes display significant AChE inhibitory activity comparable to reference drugs [9,34]. However, as previously discussed, these compounds have notable disadvantages due to their interactions with multiple biological targets, which may lead to undesirable off-target effects [28].

Among the evaluated DAAs, only cheilocline H (**9**) inhibited more than 50% of AChE activity (51.5%), exhibiting an efficacy comparable to that of its triterpenoid precursor pristimerin (**10**). In contrast, the adduct retusonine (**2**), which incorporates the tingenone (**11**) triterpenoid core linked to a sesquiterpene unit, exhibited less than half of the inhibitory activity of tingenone (23.3% and 50.9%, respectively). These results indicate that AChE inhibitory activity is significantly reduced upon adduct formation involving the tingenone triterpenoid scaffold. None of the isolated metabolites exhibited inhibitory activity comparable to that of the positive control, rivastigmine (93.1%). Nevertheless, considering these results together with those reported by Vignaux et al. [35], who found that many molecules exhibited moderate AChE inhibition, typically around 10% at 100 μM, the DAAs (**3**–**7** and **9**), with the exception of cheilocline G (**8**), which showed 31.6%, can still be regarded as promising candidates for AChE inhibition, exhibiting moderate activity with inhibition values close to 50%. Since DAAs generally exhibit reduced biological activity due to their increased molecular size [14], these findings represent a significant result and position these compounds as promising candidates for further optimization through structural modifications.

## 3. Materials and Methods

### 3.1. General Information

An Anton Paar OptoTec Model MCP 150 polarimeter (Anton Paar GmbH, Ostfildern-Scharnhausen, Germany) was used to determine optical activity ${\left[\alpha \right]}_{D}^{25{}^{\circ}}$ at 25 °C using a sodium line (λ = 589 nm). The NMR experiments were recorded on Bruker Avance 500 or 600 spectrometers (Bruker, Wissembourg, France) using the pulse sequences provided by Bruker, with CDCl_3_ as solvent, and the chemical shifts are reported in δ (ppm) with TMS as the internal reference. ESI-HRMS (Electrospray Ionization–High-Resolution Mass Spectrometry) in positive mode was performed on an LCT Premier XE Micromass Electrospray spectrometer (Waters Corporation, Milford, MA, USA). Silica gel 60 (particle size 15–40 and 63–200 µm, Macherey-Nagel, Düren, Germany) and Sephadex LH-20 (Pharmacia Biotech, Uppsala, Sweden) were used for column chromatography, and silica gel 60 F_254_ (MachereyNagel) was used for analytical or preparative TLC. The spots were visualized under UV light and by heating silica gel plates sprayed with H_2_O-H_2_SO_4_-AcOH (1:4:20). All solvents and reagents were of analytical grade and were purchased from Panreac (Barcelona, Spain).

### 3.2. Plant Material

The roots of *M. chiapensis* were collected at Montecristo National Park (latitude: 14°23′39″ N, longitude: 89°23′10″ W, elevation: 1617 m) in March 2018, located in the municipality of Metapán, Santa Ana, El Salvador. The plant species was identified by Jenny Elizabeth Menjívar Cruz, curator of the herbarium at the Museo de Historia Natural de El Salvador (MUHNES). A voucher specimen has been deposited in the herbarium under the following code: J. Menjívar 4255.

### 3.3. Extraction

The roots were dried at 40 °C for 72 h in a forced-air oven (Thermo Scientific Heratherm OMH400, Waltham, MA, USA). Once dried, the bark was removed, and the rhizodermis was ground until the particle size was <2 mm, yielding a total of 650 g of plant material. The dried and ground material was extracted in a Soxhlet apparatus, using 2 L of *n*-hexane/diethyl ether (1:1) as solvent for 24 h. The combined extracts from two extractions were concentrated under reduced pressure, using a rotary evaporator at 40 °C, yielding 27.2 g of dry extract.

### 3.4. Chromatographic Fractionation and Metabolite Isolation

The dried extract was chromatographed on a silica gel column, eluted with *n*-hexane–dichloromethane mixtures of increasing polarity, to afford 66 fractions, which were then combined based on their TLC profiles into 8 subfractions (A to H). Fraction A was subjected to silica gel chromatography, using mixtures of *n*-hexane/CH_2_Cl_2_ of increasing polarity as eluent and preparative TLC to yield cheiloclines C (**5**, 10.60 mg) and G (**8**, 1.18 mg). Gel permeation chromatography on Sephadex LH-20 (MeOH as eluent) was employed as a final purification step for fraction B, in addition to silica gel column chromatography and preparative TLC. Several chromatographic steps were done over fraction B to afford morenine (**1**, 0.87 mg), retusonine (**2**, 1.62 mg), and cheiloclines A (**3**, 5.56 mg), B (**4**, 1.26 mg), C (**5**, 7.97 mg), D (**6**, 4.97 mg), F (**7**, 2.19 mg), and H (**9**, 1.18 mg). From fractions D and E, pristimerin (**10**) and tingenone (**11**) were isolated, yielding 23.3 mg and 16.3 mg, respectively.

Morenine (**1**): colorless lacquer; ${\left[\alpha \right]}_{D}^{25}$ −34.5 (*c* 0.087, CHCl_3_); ^1^H NMR (CDCl_3_, 500 MHz), δ: 6.67 (1H, dd, *J* = 2.8, 9.8 Hz, H-6), 6.56 (1H, s, H-1), 5.89 (1H, dd, *J* = 2.8, 9.8 Hz, H-7), 5.40 (1H, s, H-3′), 4.78 (1H, s, H-12′a), 4.72 (1H, s, H-12′b), 2.96 (1H, d, *J* = 14.3 Hz, H-22a), 2.86 (1H, tt, *J* = 2.7, 11.0 Hz, H-7′), 2.59 (1H, s, H-8), 2.59 (1H, d, *J* = 16.9 Hz, H-2′b), 2.51 (1H, m, H-10′), 2.43 (1H, d, *J* = 16.9 Hz, H-2′a), 2.17 (3H, s, Me-23), 2.02 (1H, m, H-20), 2.02 (1H, m, H-6′a), 2.01 (1H, m, H-11a), 2.01 (3H, s, Me-15′), 1.86 (1H, dd, *J* = 2.4, 14.6, Hz, H-12a), 1.85 (1H, m, H-8′a), 1.78 (3H, s, Me-13′), 1.77 (1H, m, H-16a), 1.75 (1H, d, *J* = 14.3 Hz, H-22b), 1.72 (1H, m, H-19a), 1.70 (1H, m, H-11b), 1.69 (1H, m, H-6′b), 1.67 (1H, m, H-19b), 1.66 (1H, m, H-9′a), 1.73 (1H, s, H-18), 1.37 (1H, m, H-8′b), 1.33 (3H, s, Me-27), 1.30 (1H, m, H-16b), 1.26 (1H, m, H-12b), 1.23 (1H, m, H-9′b), 1.15 (1H, d, *J* = 7.22 Hz, Me-14′), 1.10 (3H, s, Me-26), 1.01 (3H, d, *J* = 5.9 Hz, Me-30), 1.00 (3H, s, Me-25), 0.95 (3H, s, Me-28); ^13^C NMR (CDCl_3_, 125 MHz), δ: 214.8 (C-21), 151.9 (C-11′), 143.4 (C-3), 142.2 (C-4′), 142.1 (C-10), 141.9 (C-2), 128.2 (CH-7), 125.1 (C-5), 125.0 (CH-3′), 120.4 (C-4), 110.5 (CH-6), 108.8 (CH_2_-12′), 102.6 (CH-1), 97.1 (C-5′), 91.2 (C-1′), 51.6 (CH_2_-22), 45.8 (CH-18), 45.5 (CH_2_-2′), 43.8 (CH-10′), 42.5 (CH-8), 42.4 (CH-20), 40.8 (CH-7′), 39.2 (C-17), 38.4 (C-9), 38.4 (C-13), 38.4 (C-14), 37.1 (CH_2_-6′), 36.6 (CH_2_-8′), 35.9 (CH_2_-16), 32.7 (CH_2_-19), 31.5 (CH_2_-11), 31.5 (CH_3_-28), 30.6 (CH_2_-9′), 29.4 (CH_2_-12), 28.1 (CH_2_-15), 20.9 (CH_3_-13′), 20.5 (CH_3_-14′), 20.4 (CH_3_-27), 19.4 (CH_3_-25), 16.3 (CH_3_-26), 15.2 (CH_3_-30), 11.5 (CH_3_-15′), 10.5 (CH_3_-23); ESI-HRMS *m*/*z* 645.4284 [M+] (calcd for C_43_H_58_O_3_Na [M+Na]^+^ 645.4284).

### 3.5. Biological Assays

#### 3.5.1. Human Cancer Cell Lines

The human solid tumor cell lines employed in this study—A549 and SW1573 (non-small-cell lung carcinoma), HBL-100 and T-47D (breast cancer), HeLa (cervical cancer), and WiDr (colon adenocarcinoma)—were kindly provided by collaborating institutions. Cells were cultured in RPMI 1640 medium supplemented with 5% fetal bovine serum (FBS) and 2 mM L-glutamine and maintained in 60 mm Petri dishes at 37 °C under a humidified atmosphere of 5% CO_2_ and 95% relative humidity. For routine maintenance, cultures were passed twice per week using 0.05% trypsin. The complete growth medium also included 5% heat-inactivated fetal calf serum, 100 U/mL penicillin, and 0.1 mg/mL streptomycin to ensure sterility and optimal cell growth.

#### 3.5.2. Antiproliferative Assay

Stock solutions at 40 mM were prepared by dissolving each sample in DMSO. The cytotoxicity assays were conducted following an in-house adaptation of the NCI60 screening protocol [30]. Cells were cultured as monolayers in 96-well microplates and exposed to test compounds for 48 h. The maximum concentration evaluated was 100 µM. Cisplatin and 5-fluorouracil served as positive controls.

#### 3.5.3. Label-Free Continuous Live Cell Imaging

SW1573 cells were seeded onto a 35 mm high glass-bottom μ-dish (IBIDI, Gräfelfing, Germany) at a density of 80,000 cells/dish. After 24 h, the growth medium was replaced with RPMI 1640 phenol red-free medium, and cells were treated with 1 μM of compound **10** for 20 h, using the CX-A label-free cell imaging system (Nanolive S.A., Tolochenaz, Switzerland), and the status of cell populations was recorded every 5 min. The initial field of observation was selected considering a homogeneous distribution of cells (236 μm × 236 μm).

#### 3.5.4. In Vitro AChE Inhibitory Activity

Enzymatic assays employing acetylcholinesterase (AChE) from *Electrophorus electricus* (type VI-S, EC 3.1.1.7, Sigma–Aldrich, St. Louis, MO, USA) were performed in 96-well plates based on spectrophotometric Ellman’s method [32], following adaptation [33]. For the initial screening at 100 μM, isolated compounds were dissolved in methanol at a stock concentration of 3.33 mM. The screening assays were performed at a final concentration of 100 μM. In a final volume of 250 µL, the samples were prepared at 3.33 mM in methanol, and 7.5 μL of this solution was added to each well of a 96-well plate containing 230 μL of phosphate buffer (0.1 M, pH 8), 2.5 μL of AChE at 1 U/mL in phosphate buffer (0.1 M, pH 8) with 1% bovine serum albumin (BSA, Sigma–Aldrich, Steinheim, Germany), and 5 μL of DTNB (5,5′-dithiobis-(2-nitrobenzoic acid), Sigma–Aldrich, Steinheim, Germany) at 3 mM in phosphate buffer (pH 8). After incubation under shaking at room temperature for 30 min, the reaction was initiated by adding 5 μL of AChI (acetylthiocholine iodide, Sigma–Aldrich, Steinheim, Germany) at 75 mM in distilled water. The assay was carried out in two experiments in triplicate, and absorbance was measured at 412 nm after 10 min of enzymatic reaction, using a spectrophotometer (Thermo Scientific, Varioskan Lux model). For negative controls, the compound solution was replaced with sodium phosphate buffer (0.1 M, pH 8), while MeOH was used as the blank for the test samples, and rivastigmine was used as a positive control.

Enzyme activity was calculated as an inhibition percentage based on the absorbance value—Inhibition (%) = 100 − (Absorbance of the sample or standard × 100)/Absorbance of the blank—from which the absorbance of the enzyme-free control has been subtracted. The results are presented as means of three independent assays ± SD.

## 4. Conclusions

The current study reports on our efforts in the search for new bioactive metabolites from plants. Thus, the isolation of a new Diels–Alder adduct, composed of a *nor*-triterpenophenol and a bicyclic guaiane-type sesquiterpene linked through a 1,4-dioxane bridge linkage, from *Maytenus chiapensis* root bark is reported. In addition, the isolation and identification of eight known adducts, together with their QMT precursors, reinforces the occurrence of these complex molecules, whose hypothetical biosynthesis involves Diels–Alderase enzymes, and they are considered chemotaxonomic markers of the Celastraceae family.

Evaluation of the isolated adducts and their triterpene precursors, pristimerin and tingenone, revealed that the last ones exhibited potent cytotoxicity across all cell lines tested, supporting previous work on the potential of QMTs as anticancer agents. In particular, pristimerin, whose cytotoxicity on the SW1573 (non-small-cell lung) cell line is reported for the first time, was more effective than the known chemotherapeutic drugs cisplatin (CDDP) and 5-fluorouracil (5-FU), and continuous live cell imaging proposed apoptosis induction and vacuole formation as the main modes of action, with a potential adaptive response that might limit the pro-apoptotic potential. Moreover, the assayed adducts showed moderate-to-low potency, likely due to their higher molecular weight and greater structural complexity relative to the QMT congeners.

Moreover, evaluation of the isolated compounds by their acetylcholinesterase inhibitory effect indicated that cheiloclines B (**4**), C (**5**), D (**6**), F (**7**), and H (**9**) exhibited moderate-to-strong inhibition, reaching up to approximately 50%. These findings suggest their potential as lead structures for further optimization in the development of AChE-targeting agents, therapeutically relevant in neurodegenerative disorders or neuromuscular dysfunctions.

The present work reinforces the potential of Celastraceae species as a source of unique and complex compounds and enhances our understanding of their therapeutic potential.

## Figures and Tables

**Table 1:** NMR spectroscopic data (500 MHz, CDCl_3_) for morenine (**1**).

	δ_C_ ^a^, Type	δ_H_ (*J* in Hz)	HMBC ^b^		δ_C_ ^a^, Type	δ_H_ (*J* in Hz)	HMBC ^b^
1	102.6, CH	6.56, s	5	23	10.5, CH_3_	2.17, s	4, 5, 3
2	141.9, C			25	19.4, CH_3_	1.00, s	8, 9, 11
3	143.4, C			26	16.3, CH_3_	1.10, s	13, 14, 15
4	120.4, C			27	20.4, CH_3_	1.33, s	18, 13, 14
5	125.1, C			28	31.5, CH_3_	0.95, s	22, 18, 17, 16
6	110.5, CH	6.67, dd (2.8, 9.8)	5, 10	30	15.2, CH_3_	1.01, d (5.9)	21, 20, 19
7	128.2, CH	5.89, dd (2.8, 9.8)		1′	91.2, C		
8	42.5, CH	2.59, s		2′a	45.5, CH_2_	2.43, d (16.9)	
9	38.4, C			2′b		2.59, d (16.9)	
10	142.1, C			3′	125.0, CH	5.40, s	2′, 1′
11a	31.5, CH_2_	2.01, m		4′	142.2, C		
11b		1.70, m		5′	97.1, C		
12a	29.4, CH_2_	1.86, dd (2.4, 14.6)		6′a	37.1, CH_2_	2.02, m	
12b		1.26, m		6′b		1.69, m	
13	38.4, C			7′	40.8, CH	2.86, tt (2.7, 11.0)	
14	38.4, C			8′a	36.6, CH_2_	1.85, m	
15	28.1, CH_2_			8′b		1.37, m	
16a	35.9, CH_2_	1.77, m		9′a	30.6, CH_2_	1.66, m	
16b		1.30, m		9′b		1.23, m	
17	39.2, C			10′	43.8, CH	2.51, m	
18	45.8, CH	1.73, s		11′	151.9, C		
19a	32.7, CH_2_	1.72, m		12′a	108.8, CH_2_	4.78, s	
19b		1.67, m		12′b		4.72, s	
20	42.4, CH	2.02, m		13′	20.90, CH_3_	1.78, s	7′, 12′, 11′
21	214.8, C			14′	20.5, CH_3_	1.15, d (7.22)	1′, 10′, 9′
22a	51.6, CH_2_	2.96, d (14.3)		15′	11.5, CH_3_	2.01, s	
22b		1.75, d (14.3)					

^a^ Data are based on edited HSQC and HMBC experiments; ^b^ selected HMBC correlations.

**Table 2:** Antiproliferative activity ^a^ (GI_50_ µM *±* SD) ^b^ of the isolated compounds ^c^.

Compound	A549	HeLa	MIA Paca-2	SW1573	T-47D	WiDr
Morenine (**1**)	78 ± 38	NA ^d^	79 ± 37	91 ± 15	89 ± 19	85 ± 26
Retusonine (**2**)	64 ± 22	NA ^d^	44.0 ± 2.1	84 ± 22	85 ± 26	NA ^d^
Cheilocline G (**8**)	26.7 ± 2.2	35 ± 15	38 ± 11	37 ± 9	44 ± 11	71 ± 22
Pristimerin (**10**)	0.9 ± 0.3	0.15 ± 0.01	0.47 ± 0.06	0.24 ± 0.07	0.61 ± 0.14	0.39 ± 0.13
Tingenone (**11**)	1.5 ± 0.1	2.1 ± 0.9	1.5 ± 0.4	2.8 ± 1.0	16.4 ± 6.6	1.6 ± 0.4
Cisplatin ^e^	4.9 ± 0.2	1.8 ± 0.5	1.6 ± 0.3	2.7 ± 0.4	16.8 ± 3.3	23.0 ± 4.3
5-Fluorouracil ^e^	2.2 ± 0.3	16.2 ± 4.5	8.3 ± 1.2	3.3 ± 1.2	43 ± 16	49.3 ± 6.7

^a^ A549 (non-small-cell lung), HBL-100 (breast), HeLa (cervix), SW1573 (non-small-cell lung), T-47D (breast), and WiDr (colon) tumor cell lines. ^b^ Values represent mean of GI_50_ ± standard deviation (SD) of at least three independent experiments. ^c^ Cheiloclines B and H were inactive against all tested cell lines, whereas cheiloclines A, C, D, and F could not be evaluated owing to limited solubility under the experimental conditions. ^d^ NA: not active (GI_50_ ≥ 100 µM). ^e^ Cisplatin and 5-fluorouracil were used as positive controls.

**Table 3:** Acetylcholinesterase inhibitory activity ^a^ of the isolated compounds.

Compound	AChE Inhibition at 100 µM
Retusonine (**2**)	23.3 ± 2.8
Cheilocline A (**3**)	42.3 ± 6.3
Cheilocline B (**4**)	46.0 ± 1.5
Cheilocline C (**5**)	46.9 ± 2.3
Cheilocline D (**6**)	43.4 ± 3.4
Cheilocline F (**7**)	45.6 ± 2.5
Cheilocline G (**8**)	31.6 ± 1.6
Cheilocline H (**9**)	51.5 ± 1.4
Pristimerin (**10**)	64.3 ± 3.9
Tingenone (**11**)	50.9 ± 4.5
Rivastigmine ^b^	93.1 ± 3.5

^a^ Expressed as percentage inhibition (mean ± standard deviation). ^b^ Rivastigmine was used as the reference standard.

## Data Availability

The original contributions presented in this study are included in the article and Supplementary Materials. Further inquiries can be directed to the corresponding author.

## Supplementary Materials

The following supporting information can be downloaded at https://www.mdpi.com/article/10.3390/ijms27073318/s1.
