# Supplementary material for: Diels–Alder Adducts from Maytenus chiapensis
Source: Int J Mol Sci. 2026 Apr 7;27(7):3318. doi: 10.3390/ijms27073318 (PMC13073600; doi:10.3390/ijms27073318)
Supplement: Supplementary file 1 [file ijms-27-03318-s001.zip › ijms-4197506-supplementary.pdf]

Supplementary material

Diels-Alder adducts from *Maytenus chiapensis*

Ulises G. Castillo[1,2], Morena L. Martínez[1], Marvin J. Núñez[1], Aday González-Bakker[3], José M. Padrón[3], Nathália Nocchi[3,4], Eduardo Hernández-Álvarez[2], Ignacio A. Jiménez[2], and Isabel L. Bazzocchi[2,*]

[1] Laboratorio de Investigación en Productos Naturales, Facultad de Química y Farmacia, Universidad de El Salvador, San Salvador 1101, El Salvador; ulises.guardado@ues.edu.sv; morena.martinez@ues.edu.sv; marvin.nunez@ues.edu.sv
[2] Instituto Universitario de Bio-Orgánica Antonio González, and Departamento de Química Orgánica, Universidad de La Laguna, Av. Astrofísico Francisco Sánchez 2, 38206 La Laguna, Spain; alu0100947311@ull.edu.es; ignadiaz@ull.edu.es; ilopez@ull.edu.es
[3] Instituto Universitario de Bio-Orgánica Antonio González, Universidad de La Laguna, Av. Astrofísico Francisco Sánchez 2, 38206; agonzaba@ull.edu.es; jmpadron@ull.edu.es; nathalianocchi@ull.edu.es
[4] Biotecnología Marina, IUBO-ULL, Unidad Asociada al IPNA-CSIC, 38206 La Laguna, Spain

Table of contents

Figure S1 1D and 2D NMR experiments, and HRMS spectrum of **morenine**

Figure S2 ^{1}H and ^{13}C NMR spectra of compound **retusonine**

Figure S3 ^{1}H and ^{13}C NMR spectra of compound **cheilocline A**

Figure S4 ^{1}H and ^{13}C NMR spectra of compound **cheilocline B**

Figure S5 ^{1}H and ^{13}C NMR spectra of compound **cheilocline C**

Figure S6 ^{1}H and ^{13}C NMR spectra of compound **cheilocline D**

Figure S7 ^{1}H and ^{13}C NMR spectra of compound **cheilocline F**

Figure S8 ^{1}H, ^{13}C NMR spectra and HMBC experiment of **cheilocline G**

Figure S9 ^{1}H and ^{13}C NMR spectra of compound **cheilocline H**

Figure S10 ^{1}H and ^{13}C NMR spectra of compound **pristimerin**

Figure S11 ^{1}H and ^{13}C NMR spectra of compound **tingenone**

Table S1 ^{1}H and ^{13}C NMR data of known compounds (**2-11**)

Video S1: Continuous live cell imaging of untreated SW1573 cells and cells exposed to compound **10** (1 μM, 20 h).

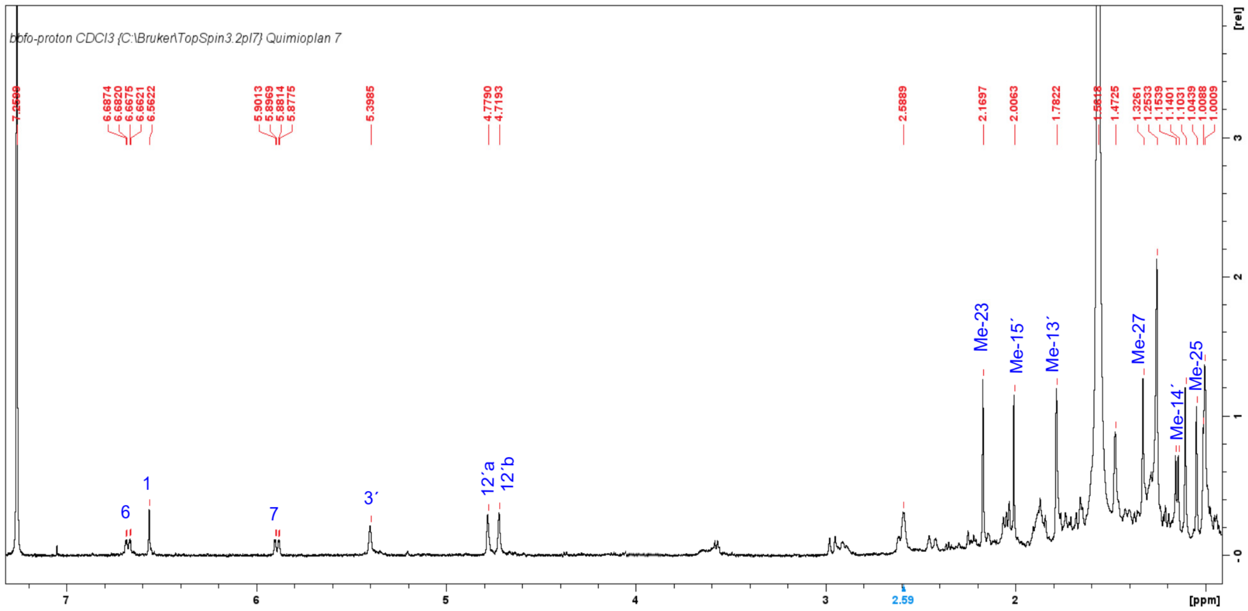

^{1}H NMR spectrum of **morenine (1)** (500 MHz, solvent $CDCl_3$)

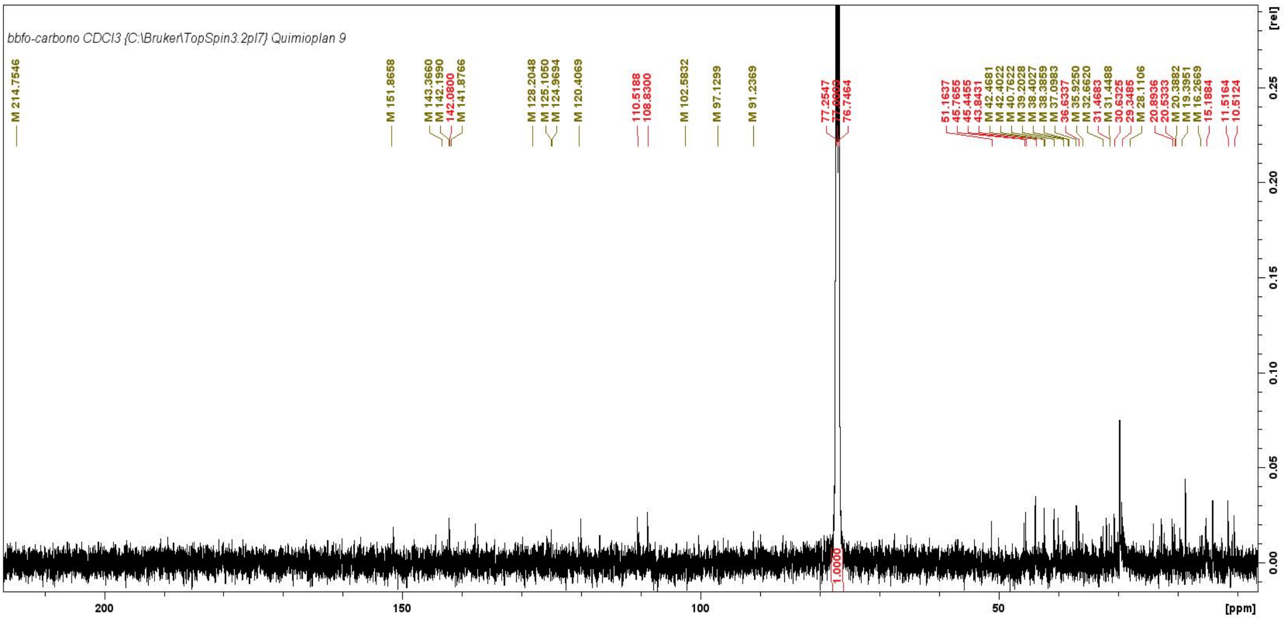

^{13}C NMR spectrum of **morenine** (**1**) (125 MHz, solvent $CDCl_3$)

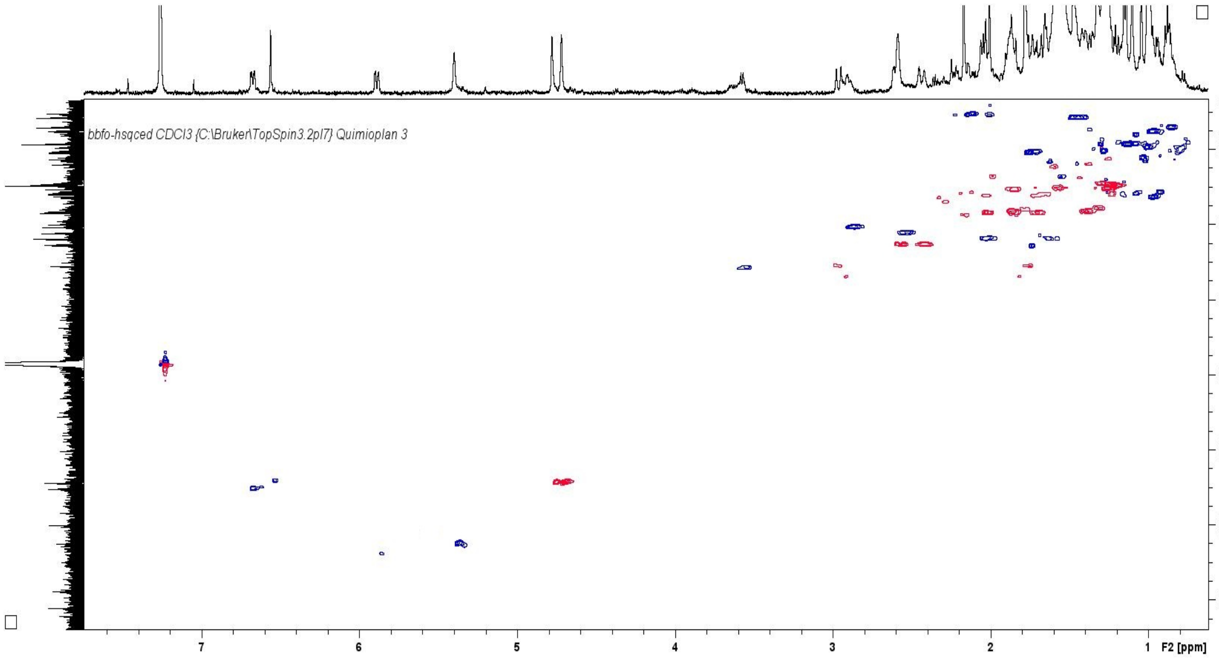

HSQC experiment of **morenine** (**1**) ^{1}H-^{13}C (600 MHz, solvent $CDCl_3$)

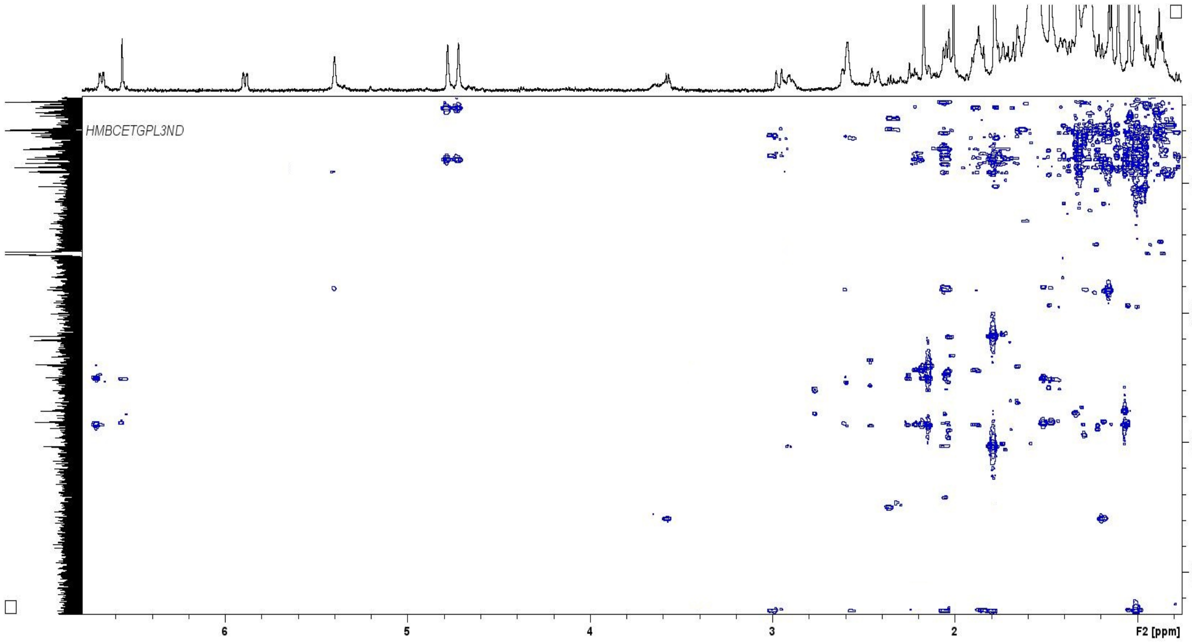

HMBC experiment of **morenine** (**1**) ^{1}H-^{13}C (600 MHz, solvent $CDCl_3$, full view)

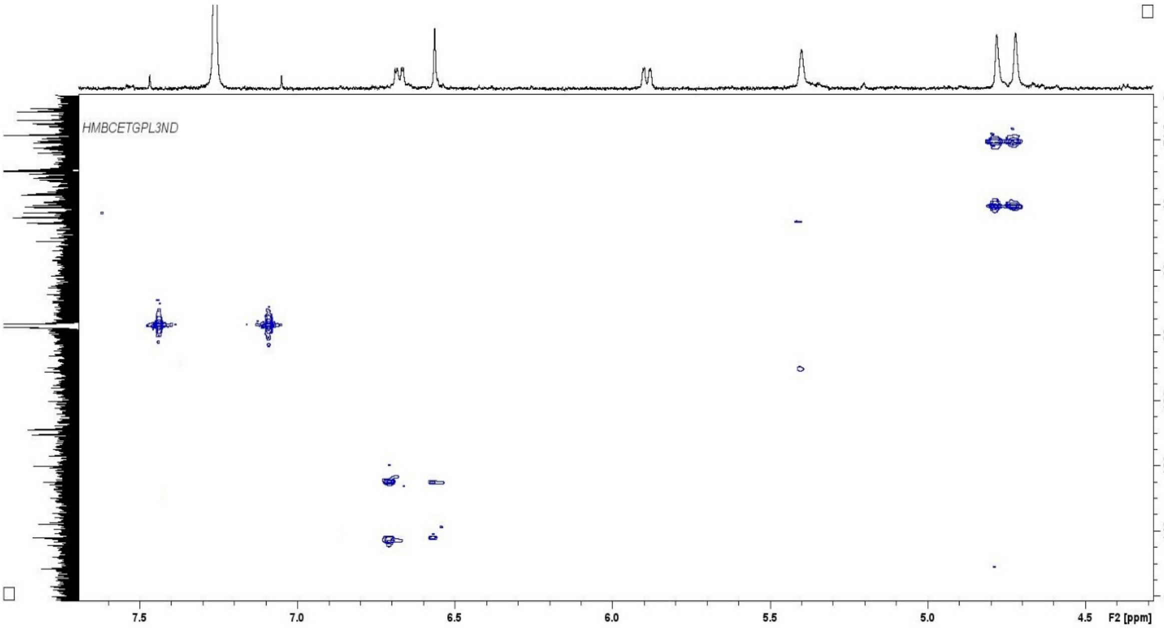

HMBC experiment of **morenine** (**1**) ^{1}H-^{13}C, selected area from 8 to 4.5ppm (600 MHz, solvent $CDCl_3$,)

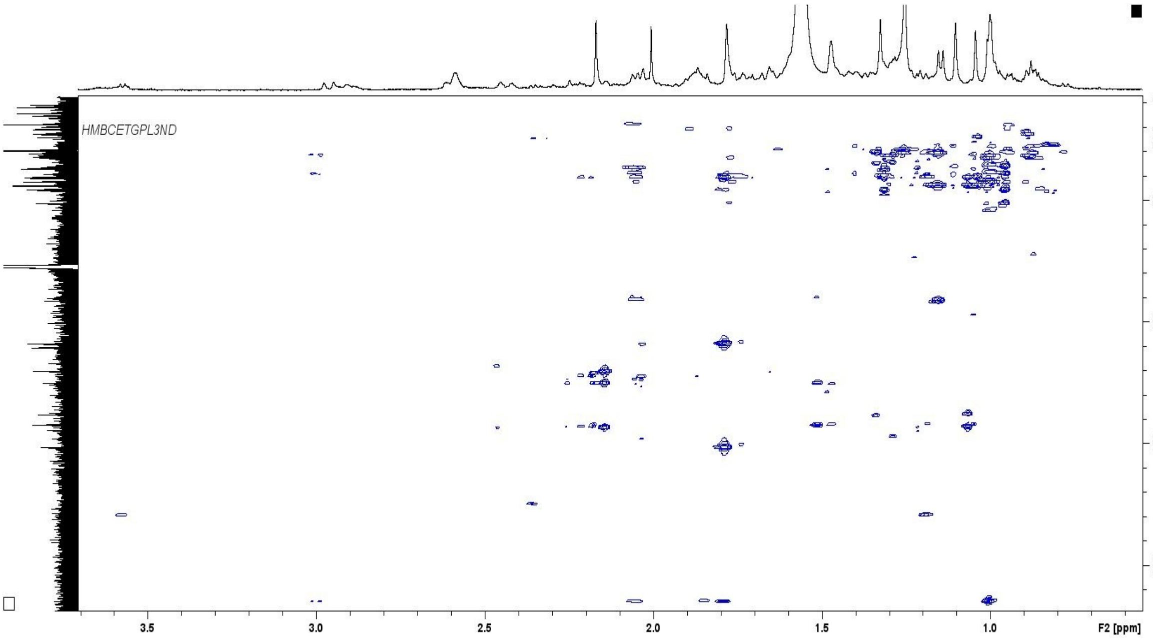

HMBC experiment of **morenine** (**1**) ^{1}H-^{13}C, selected area from 4 to 0 ppm (600 MHz, solvent $CDCl_3$)

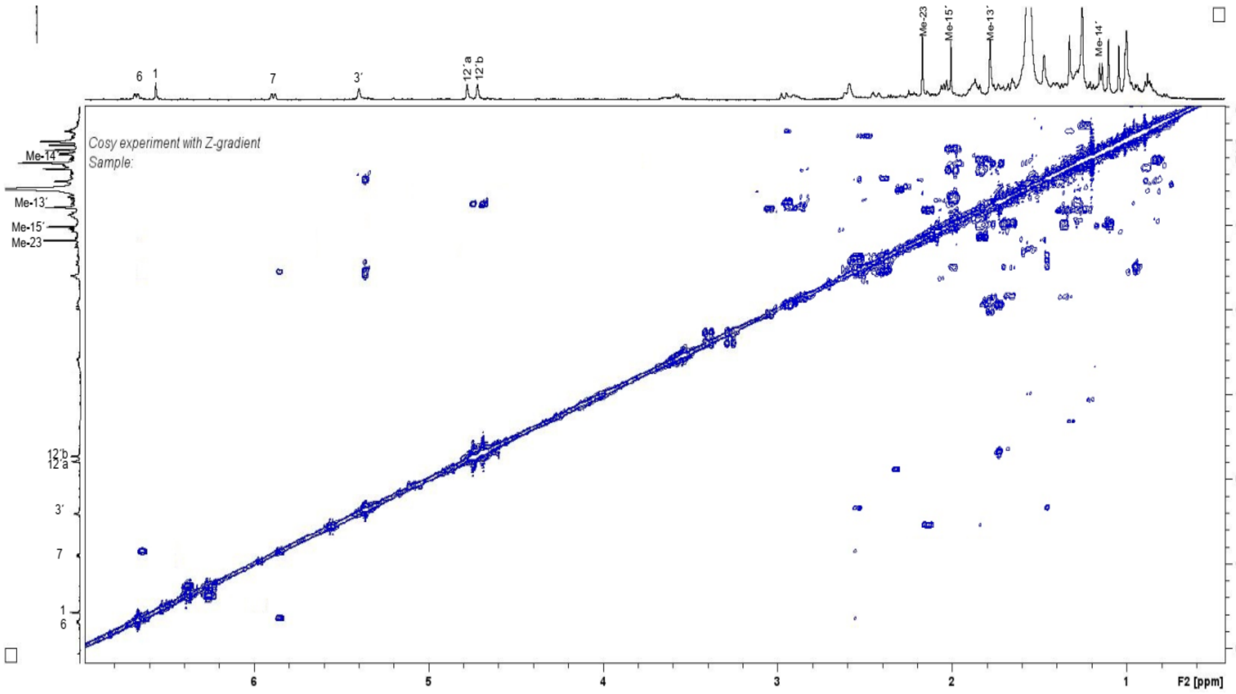

COSY experiment of **morenine** (**1**) ^{1}H-^{1}H (600 MHz, solvent $CDCl_3$)

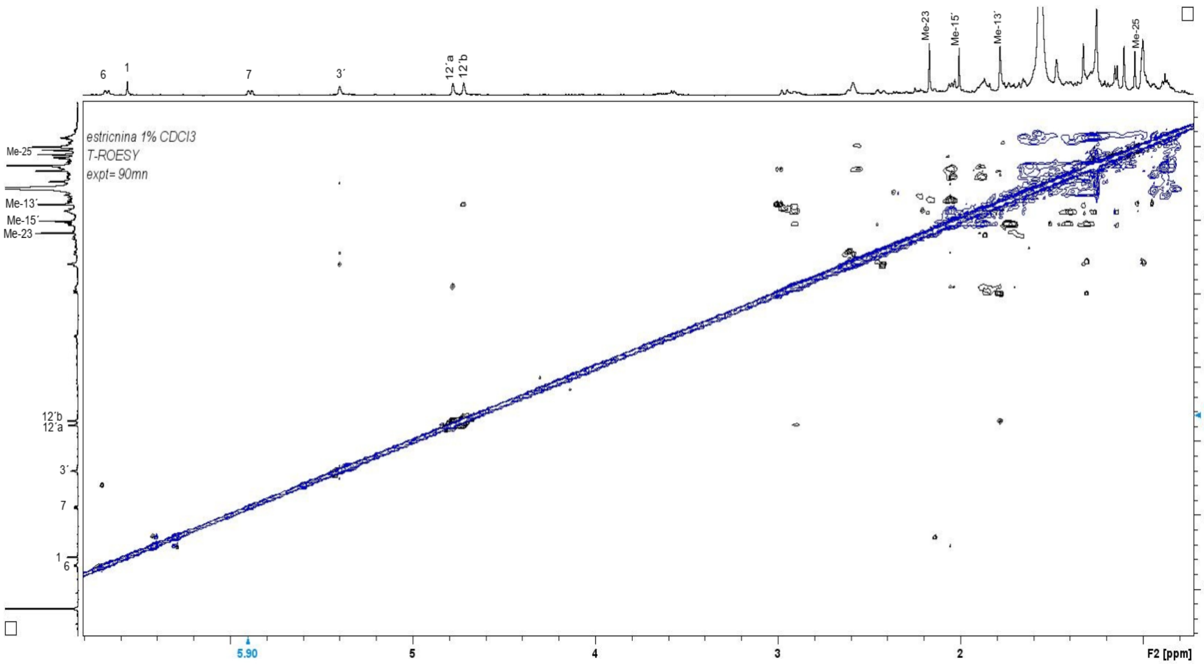

ROESY experiment of **morenine** (**1**) ^{1}H-^{1}H (600 MHz, solvent $CDCl_3$)

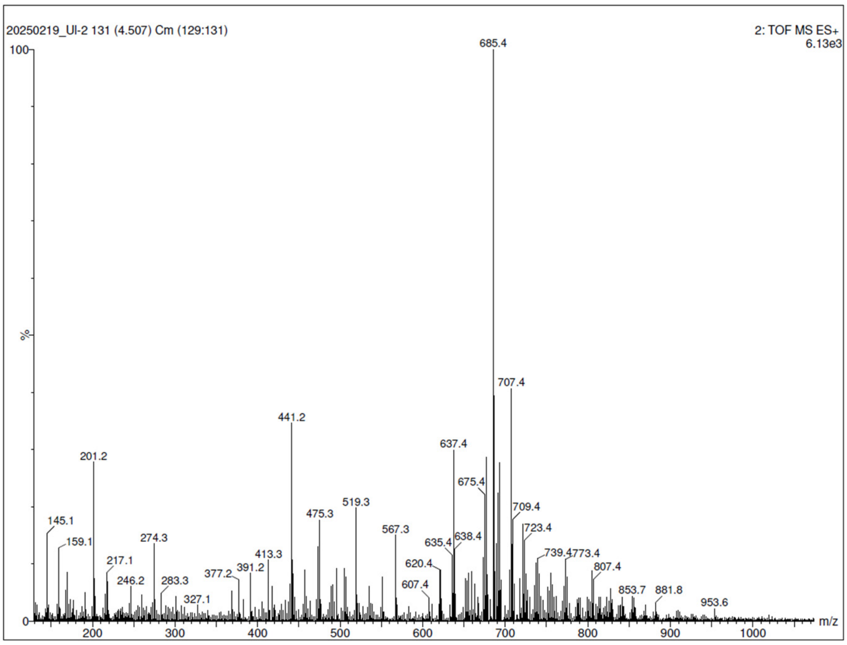

Elemental Composition Report **Page 1**

Tolerance = 5.0 PPM / DBE: min = -1.5, max = 200.0
Element prediction: Off
Number of isotope peaks used for i-FIT = 9

Monoisotopic Mass, Even Electron Ions
143 formula(e) evaluated with 3 results within limits (all results (up to 1000) for each mass)
Elements Used:
C: 0-60 H: 0-80 O: 0-6 Na: 0-2
20250219 UI-2 95 (3.255)
2: TOF MS ES+

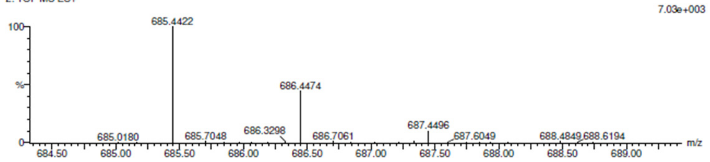

Minimum:	50.00				-1.5			
Maximum:	100.00		5.0	5.0	200.0			
Mass	RA	Calc. Mass	mDa	PPM	DBE	i-FIT	i-FIT (Norm)	Formula
685.4422	100.00	685.4420	0.2	0.3	8.5	154.9	0.2	C40 H63 O6 Na2
		685.4409	1.3	1.9	23.5	159.9	5.2	C51 H57 O
		685.4444	-2.2	-3.2	11.5	156.5	1.7	C42 H62 O6 Na

Figure S1: 1D and 2D NMR experiments, and ESI-HRMS spectrum of **morenine** (**1**).

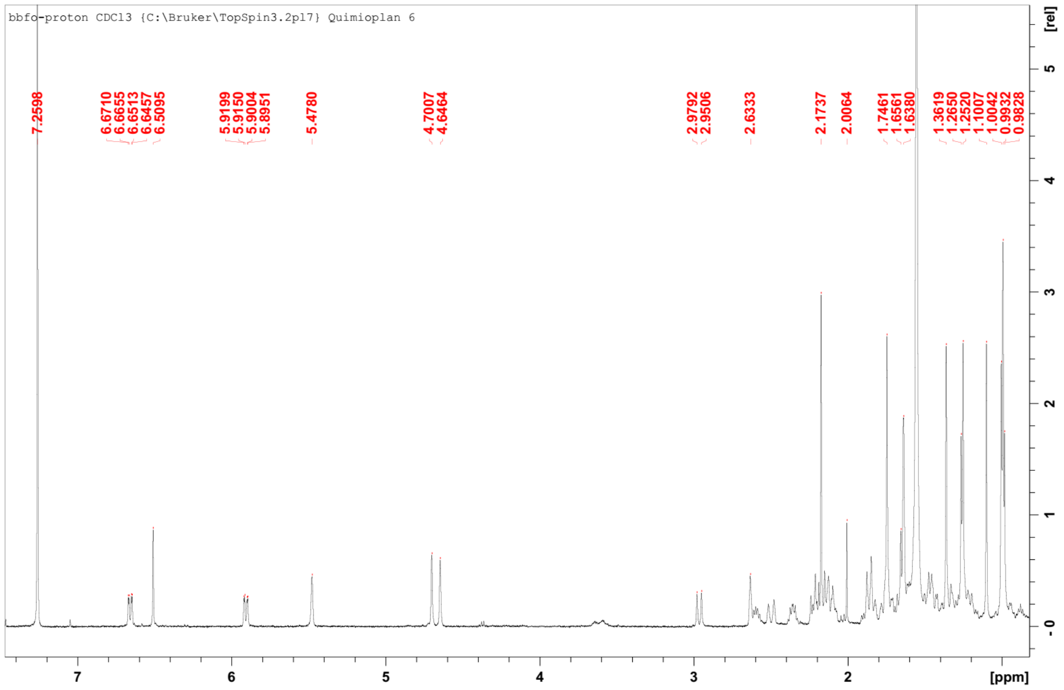

^{1}H NMR spectrum of **retusonine** (500 MHz, solvent $CDCl_3$)

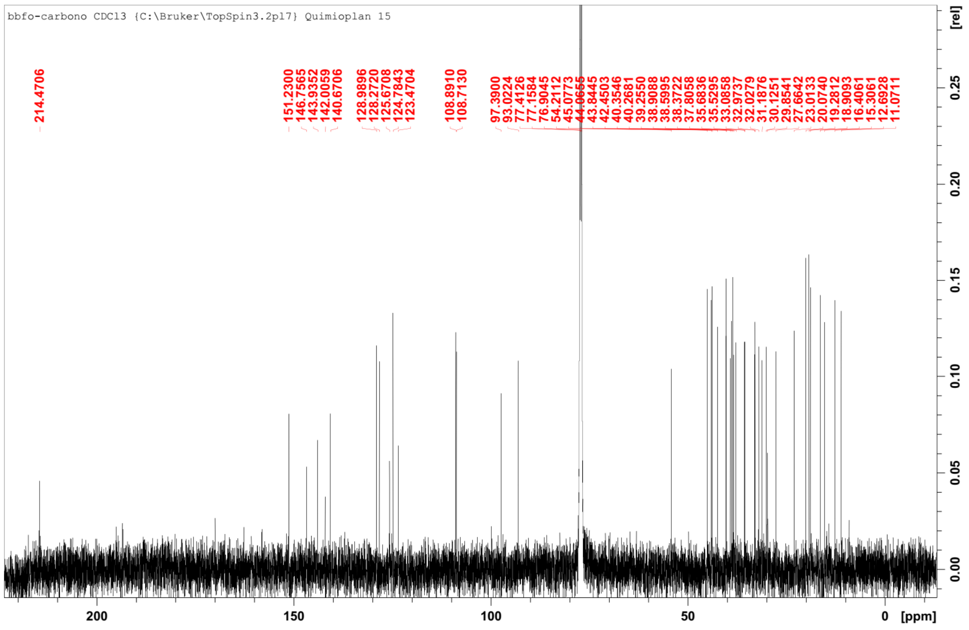

^{13}C NMR spectrum of **retusonine** (125 MHz, solvent $CDCl_3$)

Figure S2: ^{1}H and ^{13}C NMR spectra of **retusonine**

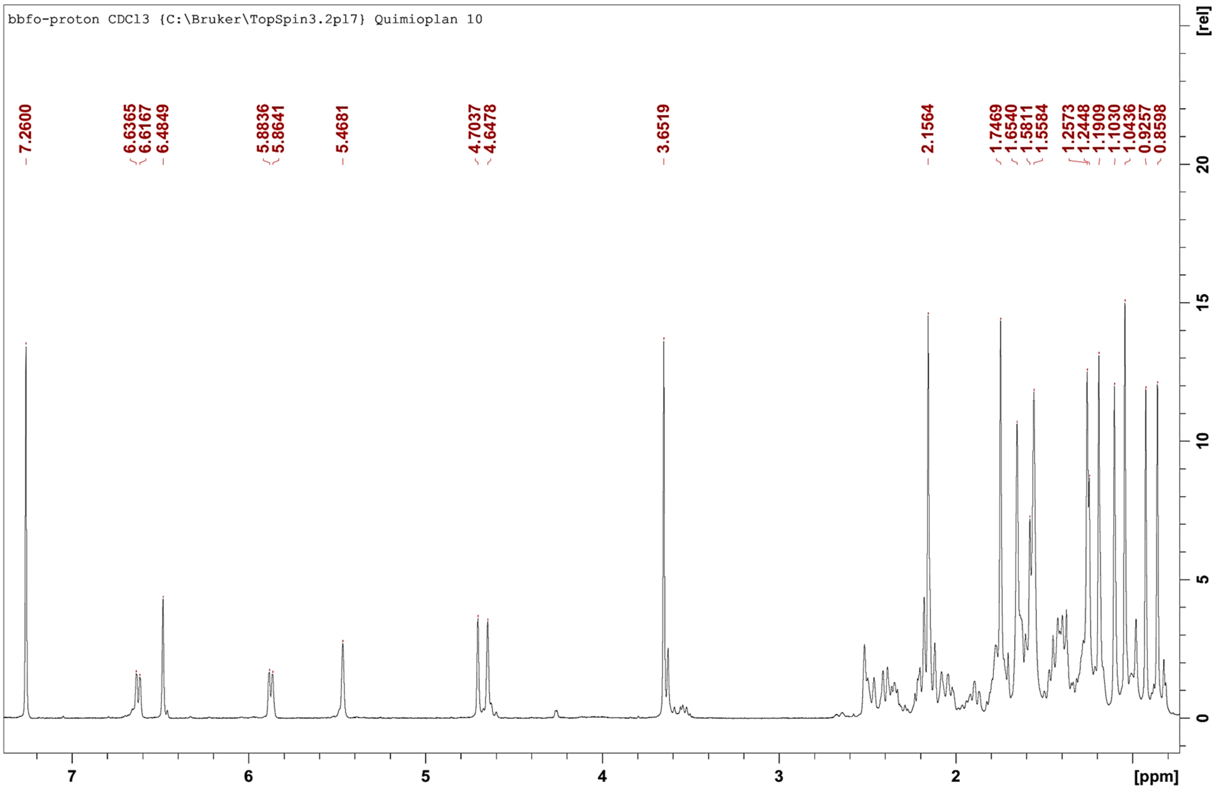

^{1}H NMR spectrum of **cheilocline A** (500 MHz, solvent $CDCl_3$)

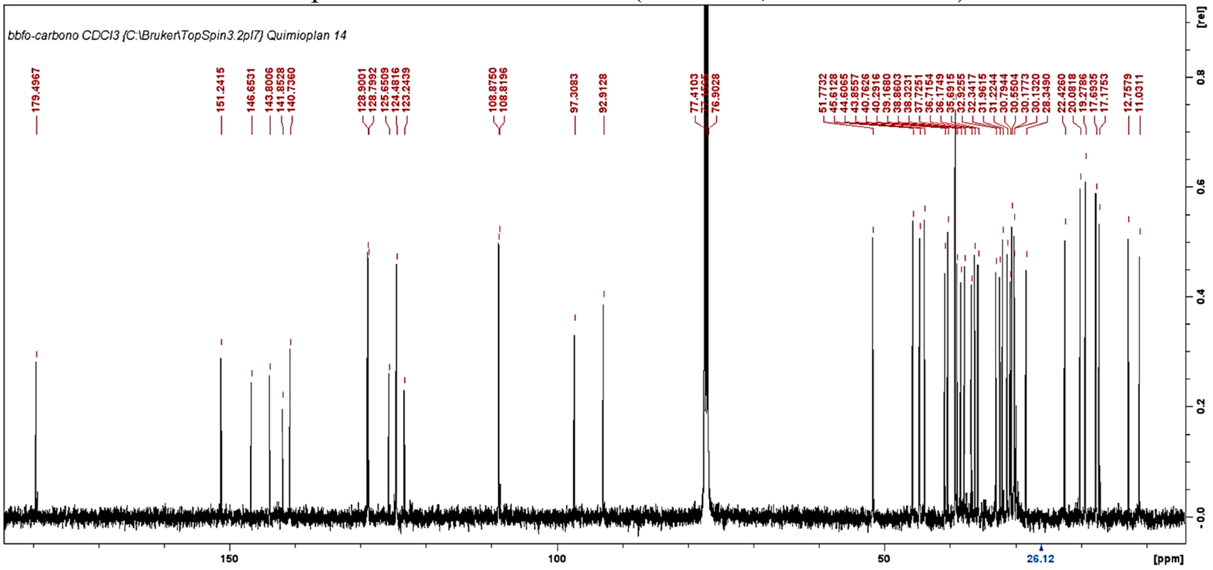

^{13}C NMR spectrum of **cheilocline A** (125 MHz, solvent $CDCl_3$)

Figure S3: ^{1}H and ^{13}C NMR spectra of **cheilocline A**

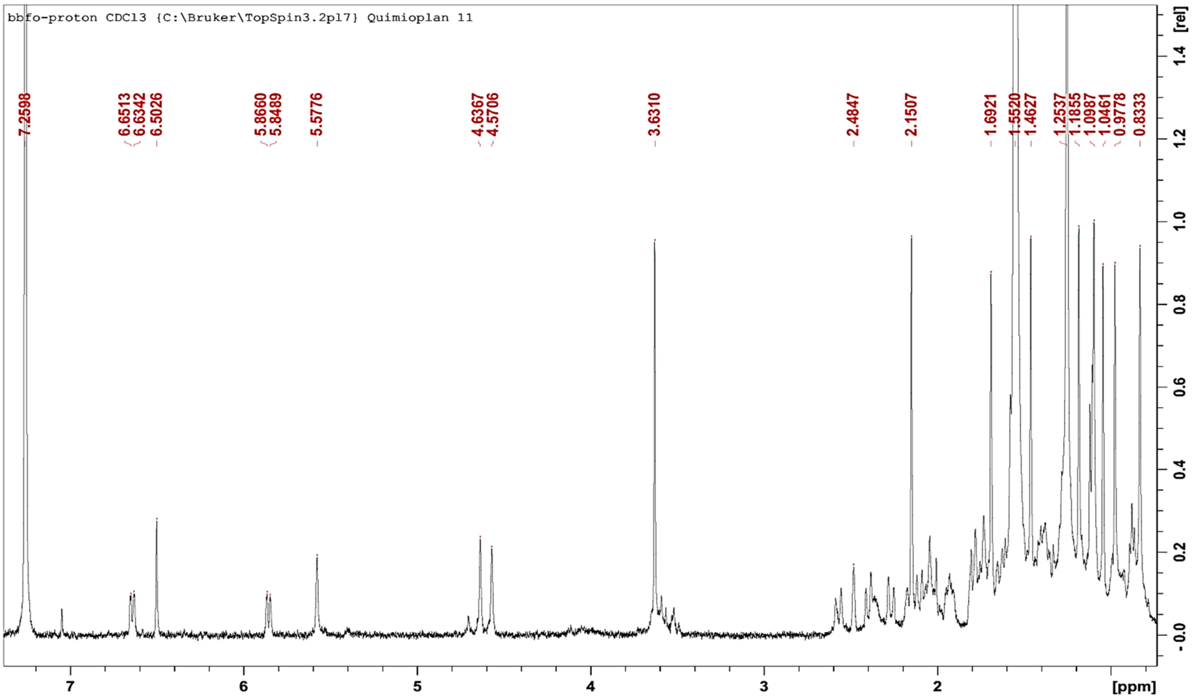

^{1}H NMR spectrum of **cheilocline B** (500 MHz, solvent $CDCl_3$)

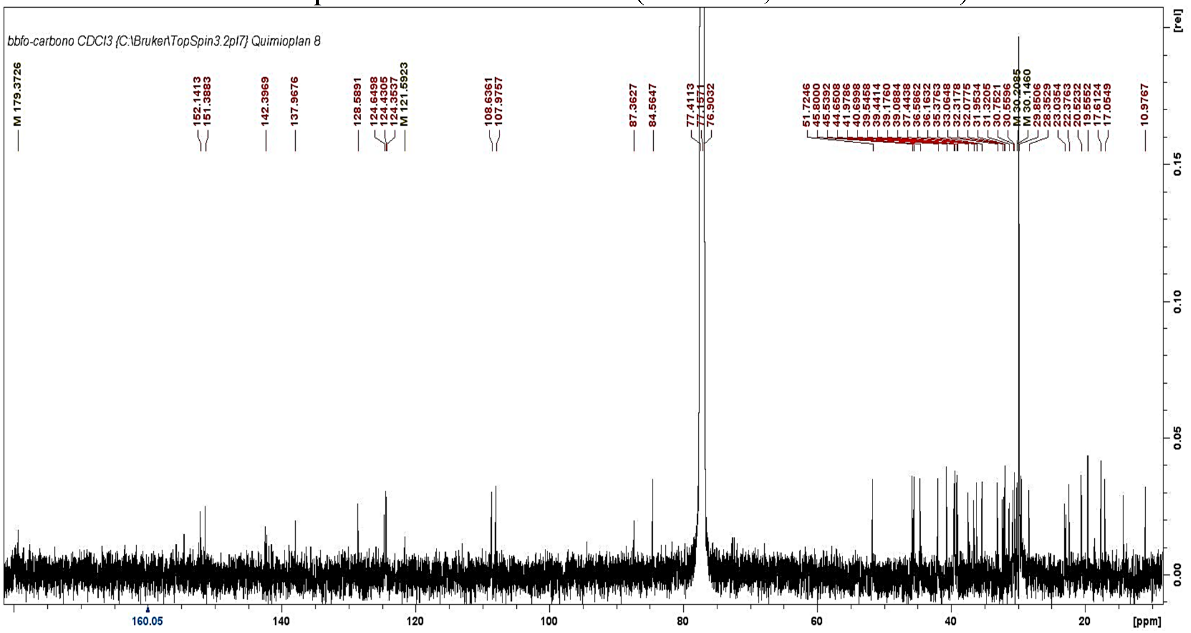

^{13}C NMR spectrum of **cheilocline B** (125 MHz, solvent $CDCl_3$)

Figure S4: ^{1}H and ^{13}C NMR spectra of **cheilocline B**

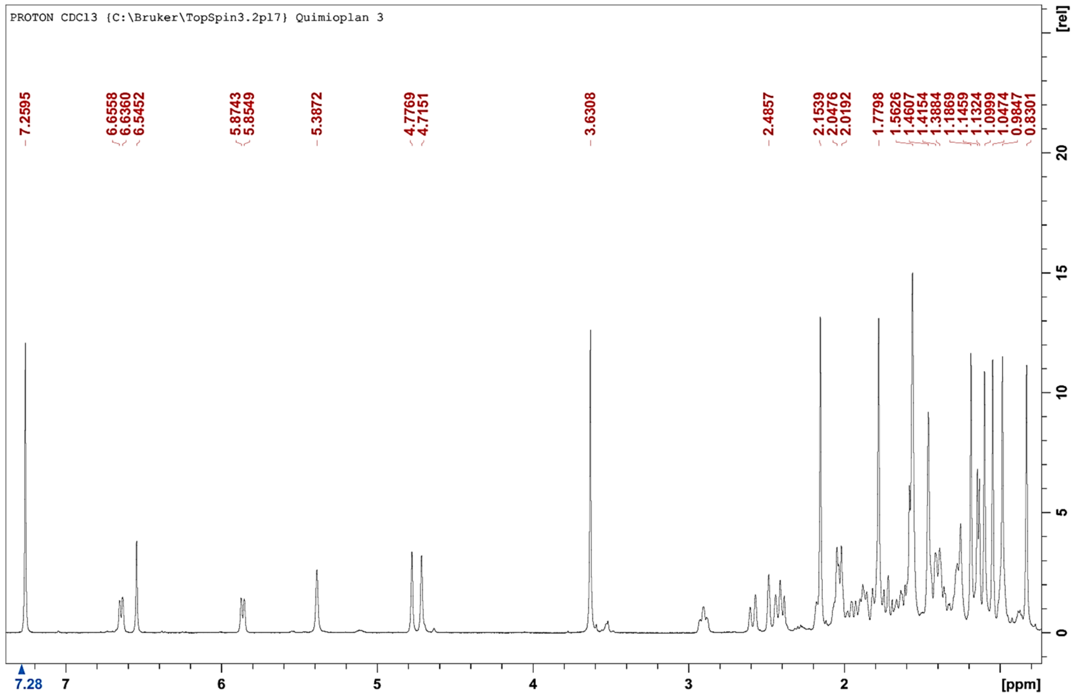

^{1}H NMR spectrum of **cheilocline C** (500 MHz, solvent $CDCl_3$)

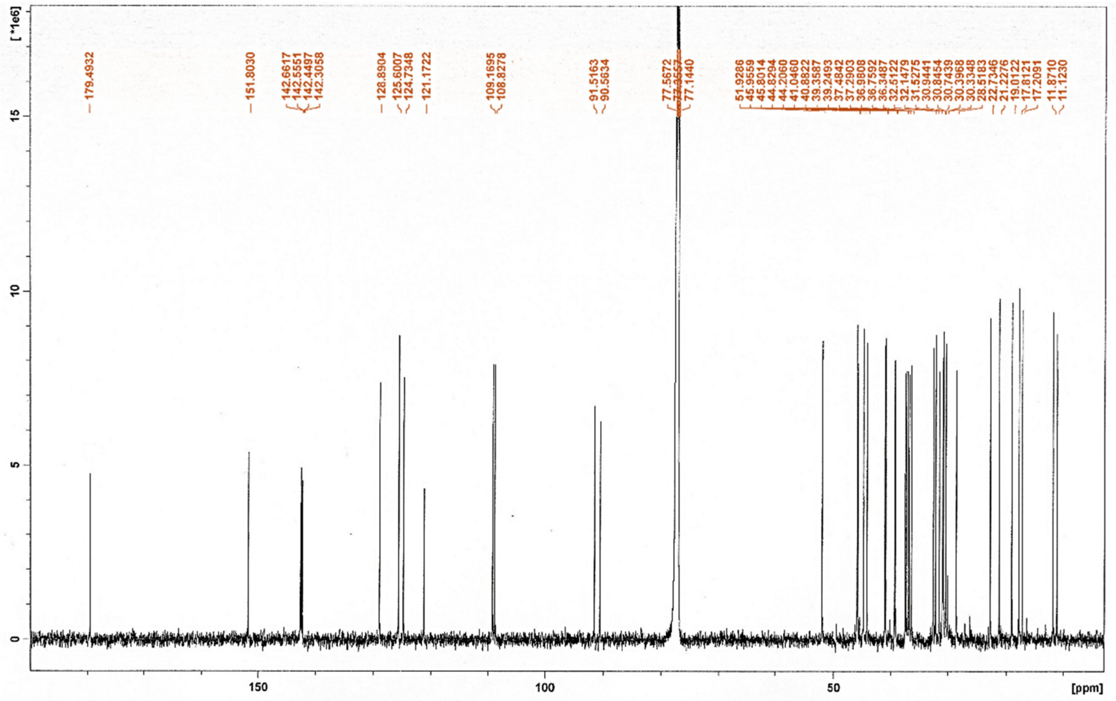

^{13}C NMR spectrum of **cheilocline C** (125 MHz, solvent $CDCl_3$)

Figure S5: ^{1}H and ^{13}C NMR spectra of **cheilocline C**

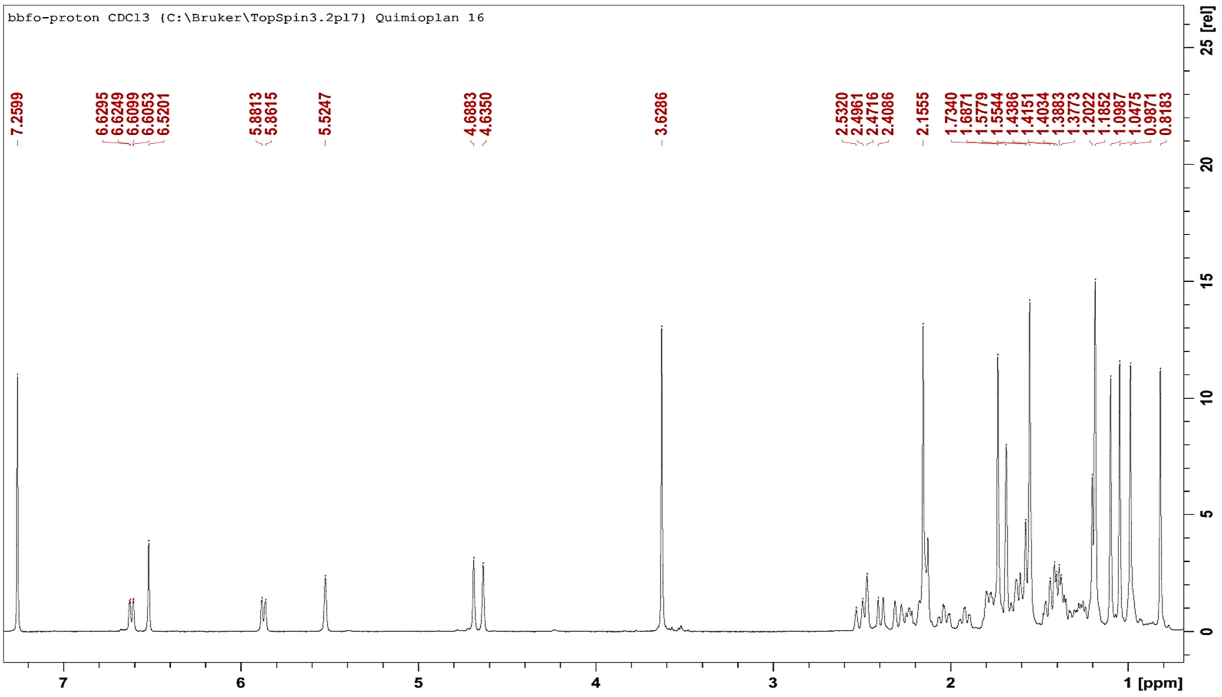

^{1}H NMR spectrum of **cheilocline D** (500 MHz, solvent $CDCl_3$)

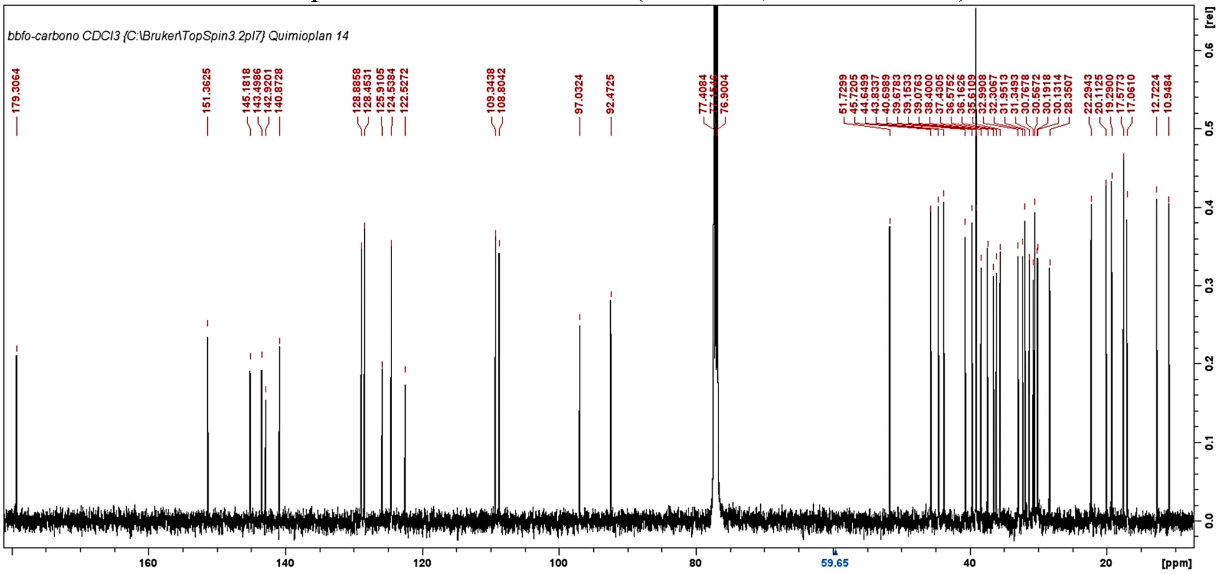

^{13}C NMR spectrum of **cheilocline D** (125 MHz, solvent $CDCl_3$)

Figure S6: ^{1}H and ^{13}C NMR spectra of **cheilocline D**

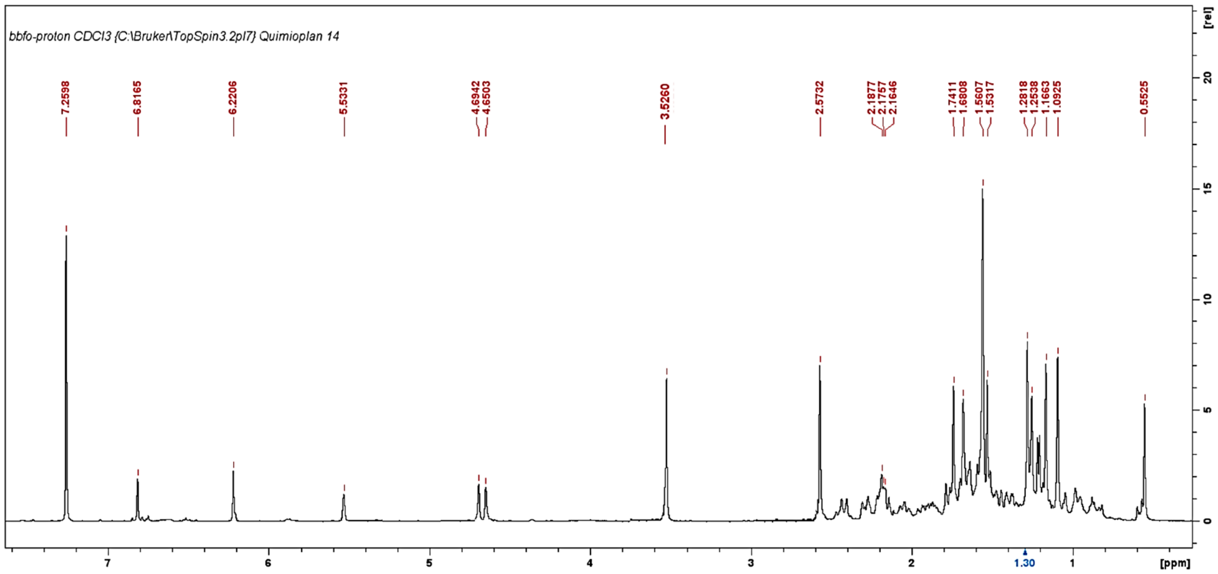

^{1}H NMR spectrum of **cheilocline F** (500 MHz, solvent $CDCl_3$)

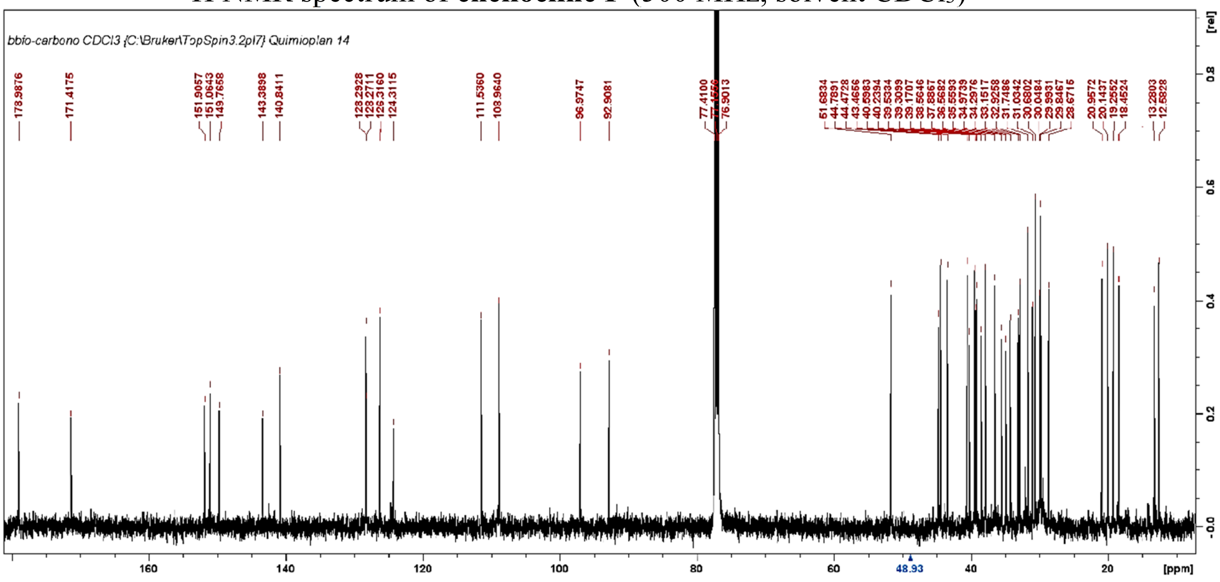

^{13}C NMR spectrum of **cheilocline F** (125 MHz, solvent $CDCl_3$)

Figure S7: ^{1}H and ^{13}C NMR spectra of **cheilocline F**

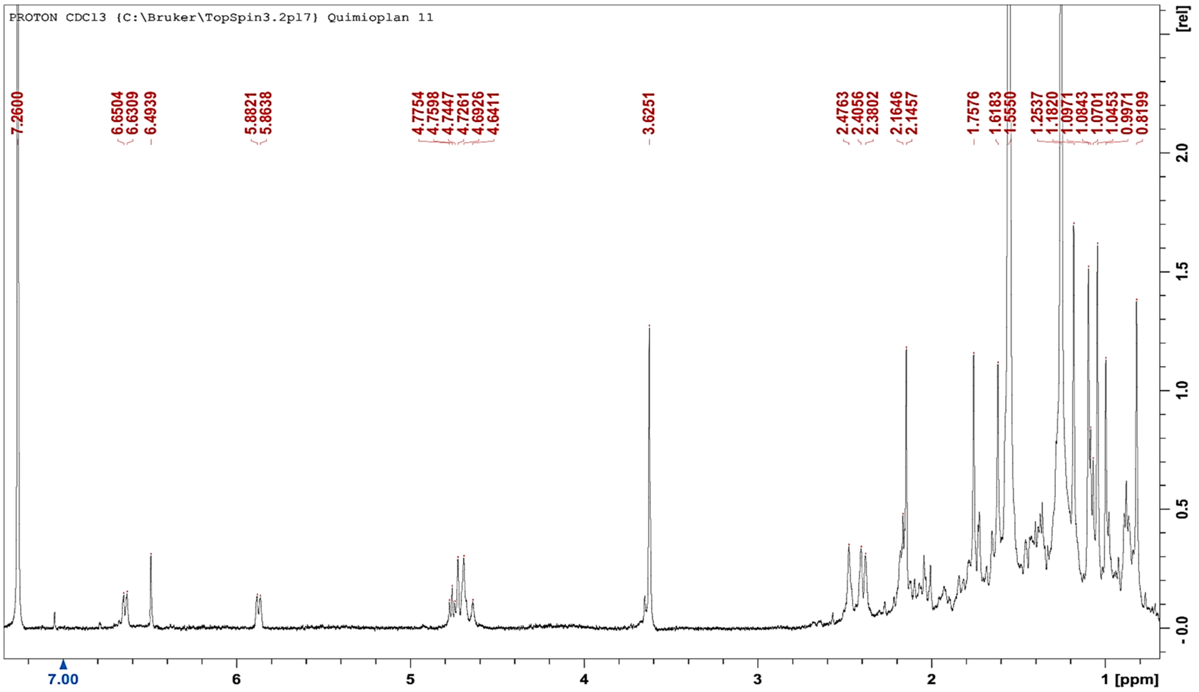

^{1}H NMR spectrum of **cheilocline G** (500 MHz, solvent $CDCl_3$)

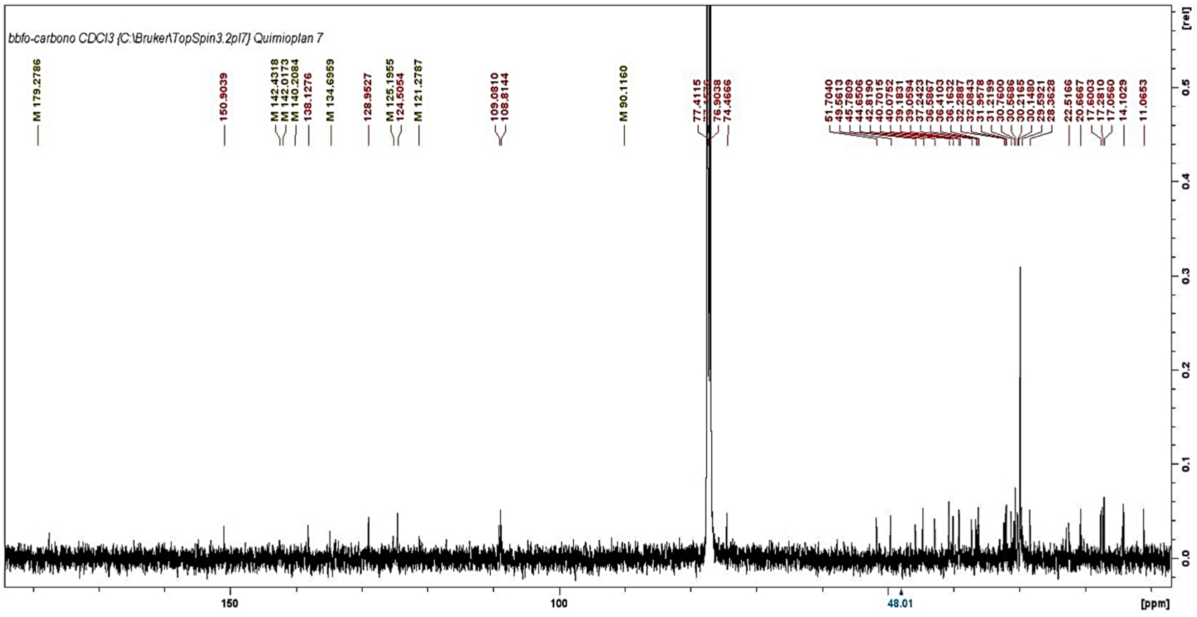

^{13}C NMR spectrum of **cheilocline G** (125 MHz, solvent $CDCl_3$)

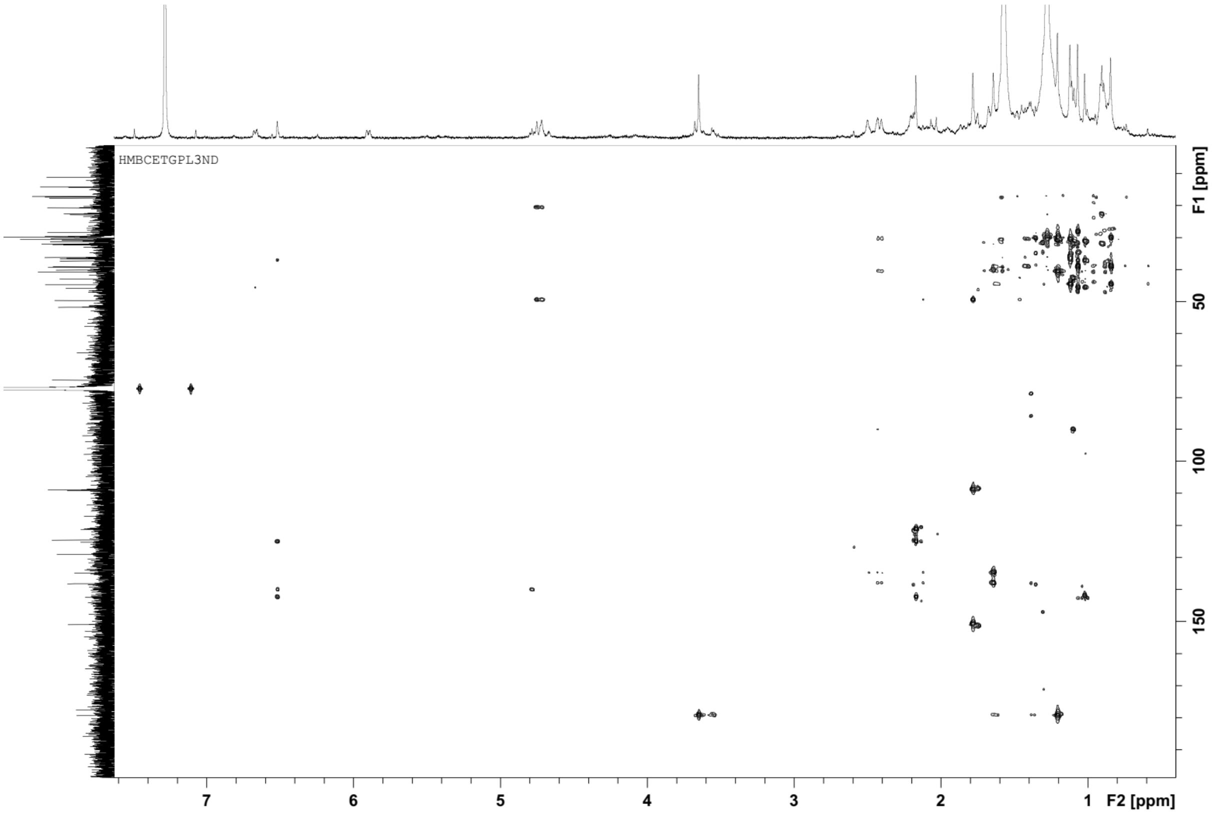

HMBC experiment of **cheilocline G** ^{1}H-^{13}C (600 MHz, solvent $CDCl_3$)

Figure S8: ^{1}H, ^{13}C NMR spectra and HMBC experiment of **cheilocline G**

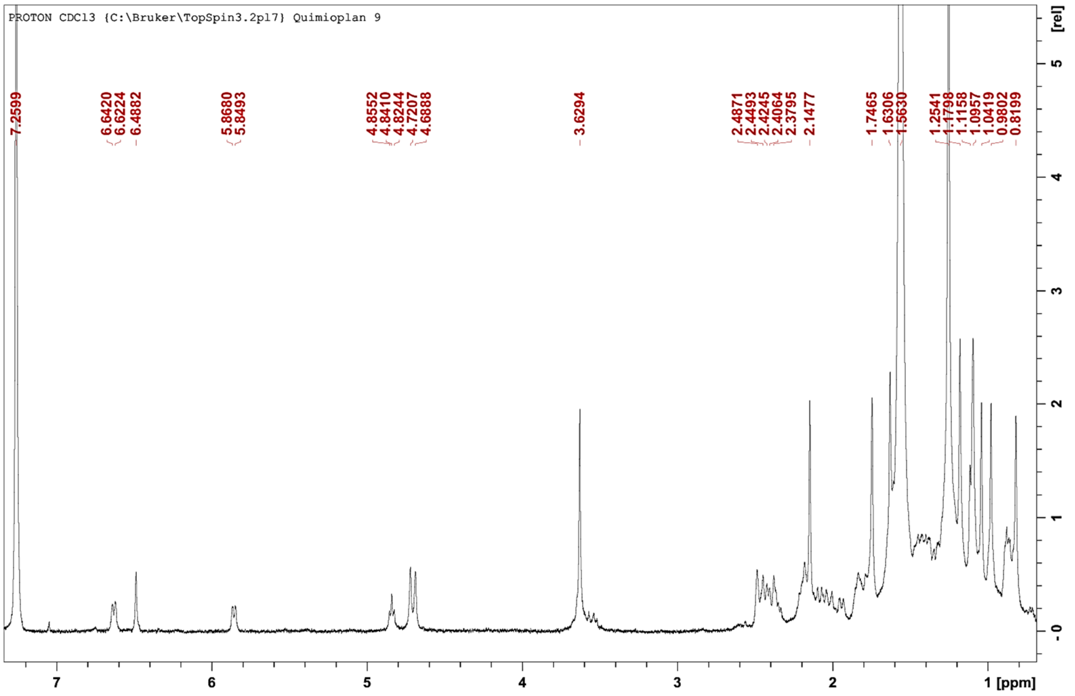

^{1}H NMR spectrum of **cheilocline H** (500 MHz, solvent $CDCl_3$)

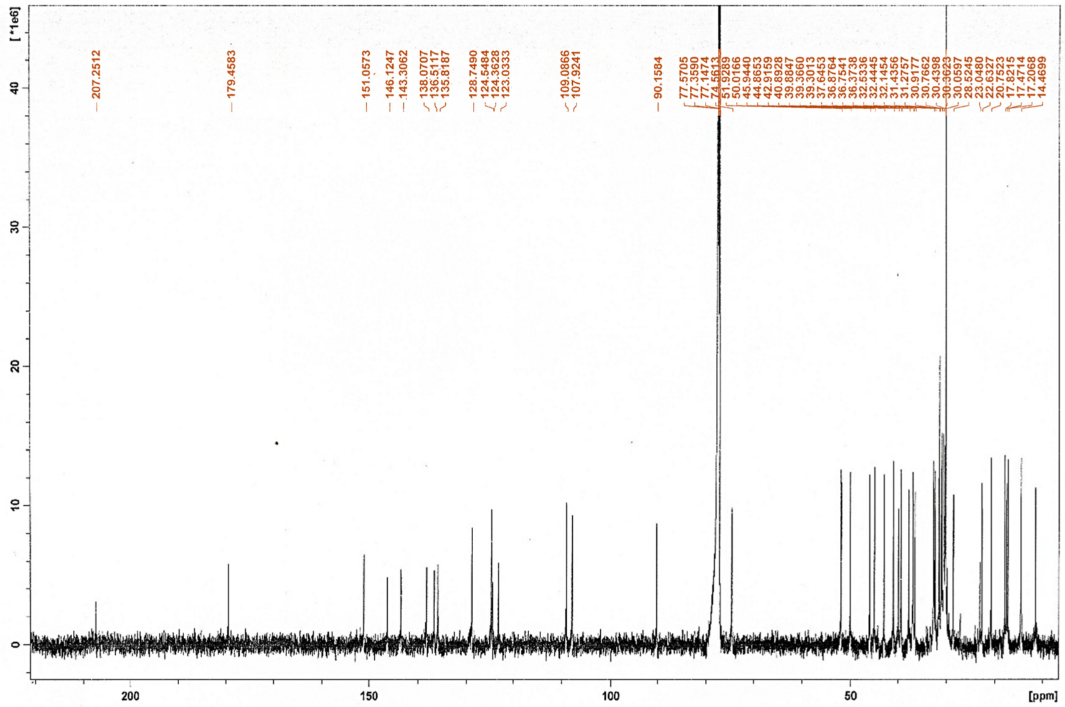

^{13}C NMR spectrum of **cheilocline H** (125 MHz, solvent $CDCl_3$)

Figure S9: ^{1}H and ^{13}C NMR spectra of **cheilocline H**

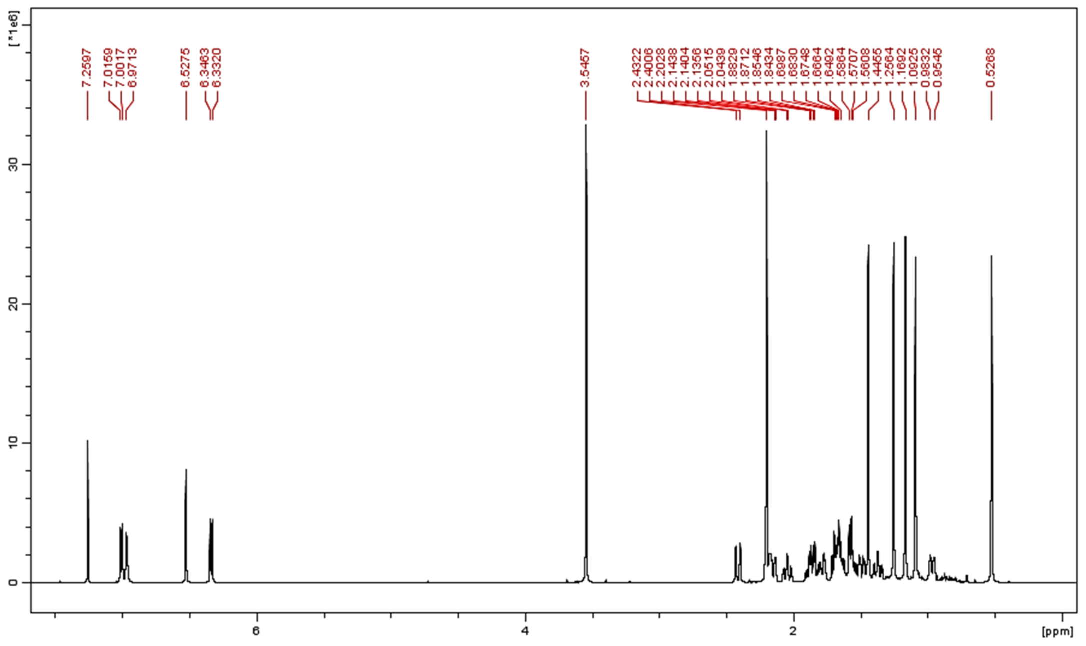

^{1}H NMR spectrum of **pristimerin** (500 MHz, solvent $CDCl_3$)

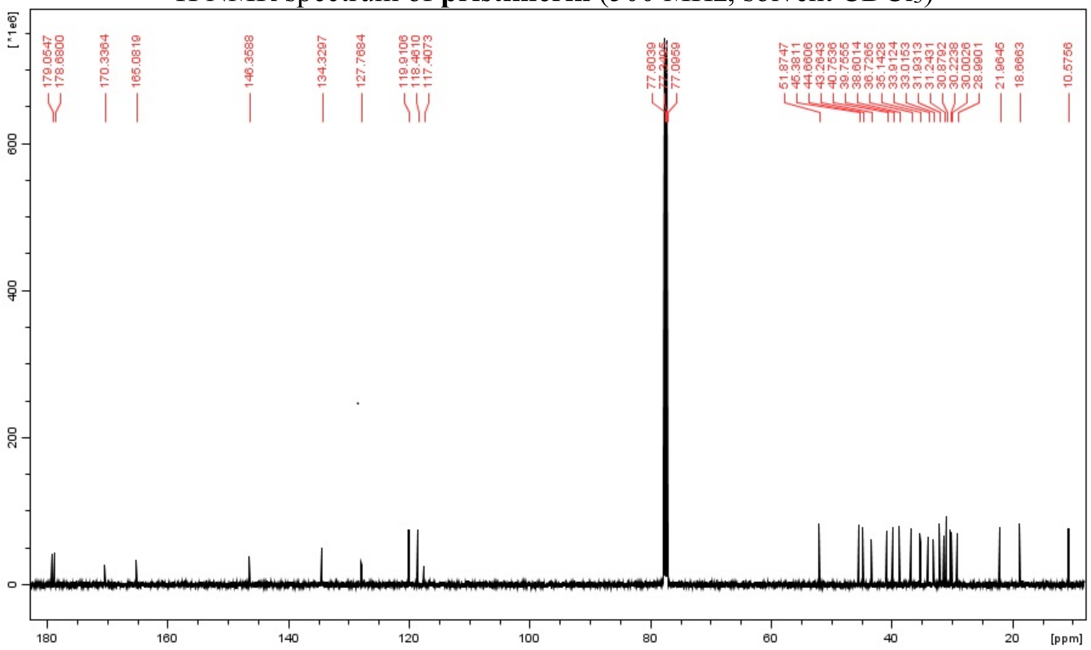

^{13}C NMR spectrum of **pristimerin** (125 MHz, solvent $CDCl_3$)

Figure S10: ^{1}H and ^{13}C NMR spectra of **pristimerin**

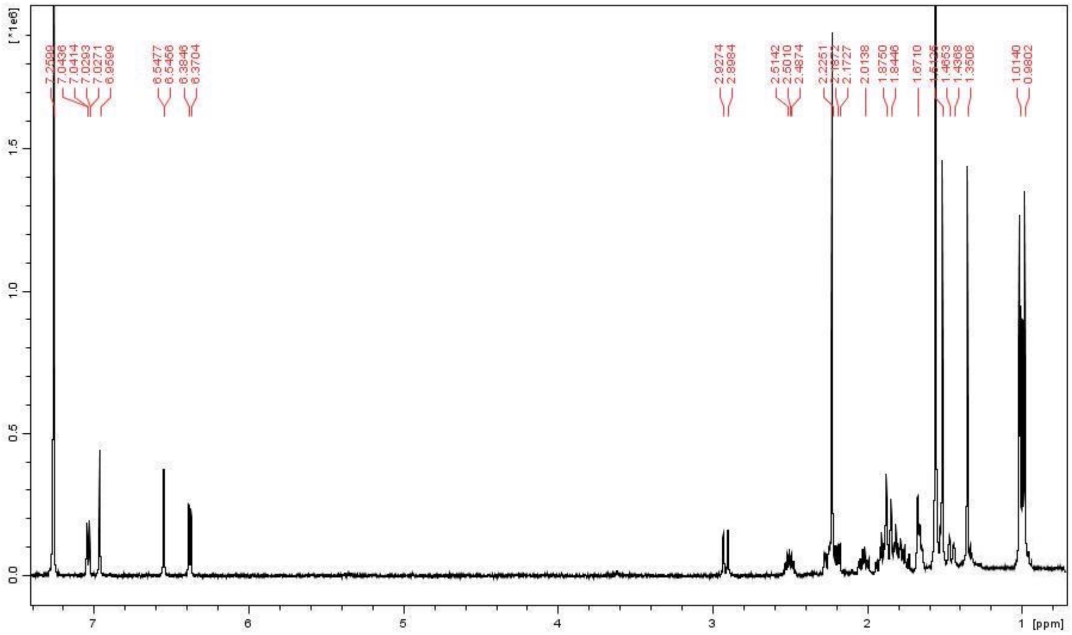

^{1}H NMR spectrum of **tingenone** (500 MHz, solvent $CDCl_3$)

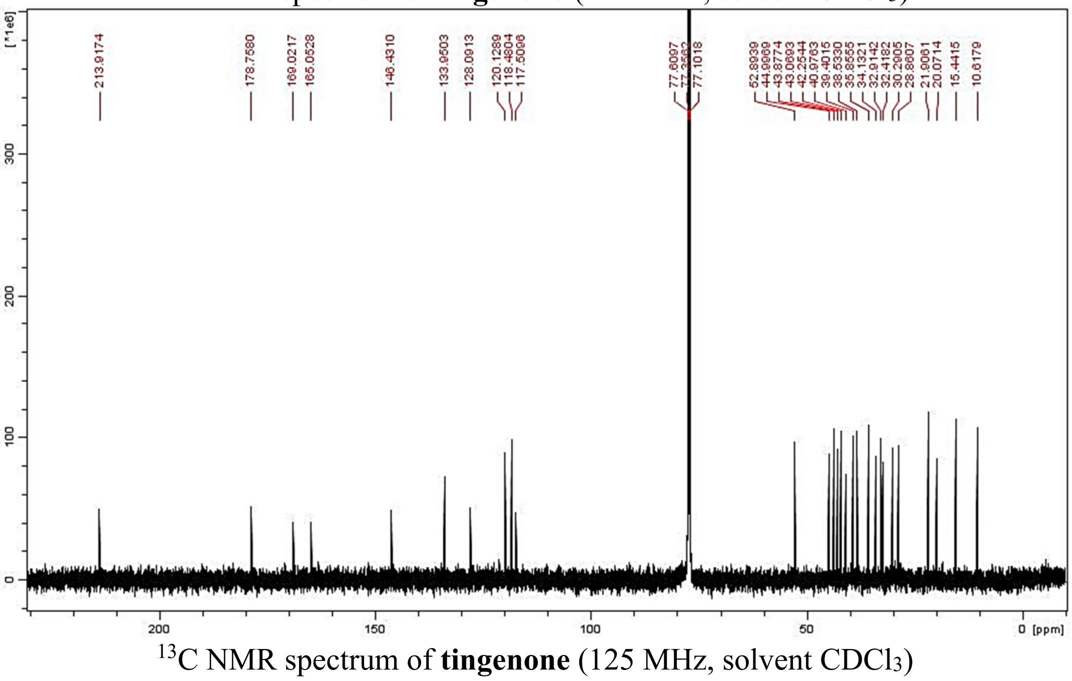

^{13}C NMR spectrum of **tingenone** (125 MHz, solvent $CDCl_3$)

Figure S11: ^{1}H and ^{13}C NMR spectra of **tingenone**

^{1}H NMR Spectroscopic Data (500 MHz, $CDCl_3$) for compounds **2-11**.

**[1]H**	(**2**): δH (*J* in Hz)	(**3**): δH (*J* in Hz)	(**4**): δH (*J* in Hz)	(**5**): δH (*J* in Hz)	(**6**): δH (*J* in Hz)	(**7**): δH (*J* in Hz)	(**8**): δH (*J* in Hz)	(**9**): δH (*J* in Hz)	(**10**): δH (*J* in Hz)	(**11**): δH (*J* in Hz)
1	6.51, 1H, s	6.48, 1H, s	6.50, 1H, s	6.55,1H, s	6.52, 1H, s	6.82, 1H, s	6.49, 1H, s	6.49, 1H, s	6.53, 1H, s	6.54, 1H, s
6	6.66, 1H, dd (2.8, 9.9)	6.62, 1H, dd (1.9, 9.9)	6.62, 1H, dd (2.9, 9.9)	6.65, 1H, dd (2.9, 9.9)	6.62, 1H, dd (2.3, 9.9)	-	6.64, 1H, dd (2.9, 9.9)	6.64, 1H, dd (2.7, 9.9)	7.00, 1H, dd (0.9, 7.2)	7.03, 1H, d (7.4)
7	5.91, 1H, dd (2.6, 9.8)	5.87, 1H, dd (1.9, 9.9)	5.86, 1H, dd (2.7, 9.9)	5.86, 1H, dd (2.7, 9.9)	5.87, 1H, dd (2.0, 9.9)	6.22, 1H, s	5.87, 1H, dd (2.8, 9.9)	5.87, 1H, dd (2.4, 9.9)	6.33, 1H, d (7.2)	6.37, 1H, d (7.2)
8	2.59, 1H, bs	2.50, 1H, bs	2.49, 1H, bs	2.49, 1H, bs	1.58, 1H, s	-	2.47, 1H, s	2.49, 1H, s		
11a	1.87, 1H, m									
11b	1.27, 1H, m									
18	2.63, 1H, bs	1.58, 1H, s	1.58, 1H, s	1.60,1H, s	1.73,1H, s	1.58, 1H, s	1.57, 1H, bs	1.58, 1H, s		
19									2.43, 2H, d (15)	
20										2.50, 1H, m (7.4)
22α	2.96, 1H, d (14.3)									2.91, 2H, d (14.3)
22β	1.82, 1H, d (14.3)									
23	2.17, 3H, s	2.15, 3H, s	2.15, 3H, s	2.16, 3H, s	2.15, 3H, s	2.57, 3H, s	2.15, 3H, s	2.14, 3H, s	2.20, 3H, s	2.20, 3H, s
25	1.01, 3H, s	0.93, 3H, s	0.98, 3H, s	0.99, 3H, s	0.99, 3H, s	1.53, 3H, s	1.00, 3H, s	0.98, 3H, s	1.45, 3H, s	1.51, 3H, s
26	1.10, 3H, s	1.04, 3H, s	1.05, 3H, s	1.05, 3H, s	1.05, 3H, s	1.28, 3H, s	1.05, 3H, s	1.04, 3H, s	1.26, 3H, s	1.35, 3H, s
27	1.36, 3H, s	0.85, 3H, s	0.83, 3H, s	0.83, 3H, s	0.82, 3H, s	0.55, 3H, s	0.82, 3H, s	0.82, 3H, s	0.53, 3H, s	1.01, 3H, s
28	0.98, 3H, s	1.10, 3H, s	1.10, 3H, s	1.10, 3H, s	1.10, 3H, s	1.09, 3H, s	1.10, 3H, s	1.10, 3H, s	1.09, 3H, s	1.26, 3H, s
30	0.99, 3H, d (5.9 Hz)	1.19, 3H, s	1.19, 3H, s	1.19, 3H, s	1.19, 3H, s	1.16, 3H, s	1.18, 3H, s	1.18, 3H, s	1.17, 3H, s	0.98, 3H, d (6)
OMe		3.65, 3H, s	3.63, 3H, s	3.63, 3H, s	3.63, 3H, s	3.53, 3H, s	3.63, 3H, s	3.63, 3H, s	3.55, 3H, s	
3´	5.48, 1H, s	5.45, 1H, bs	5.58, 1H, d (1.62)	5.39, 1H, bs	5.52, 1H, s	5.53, 1H, s	4.76, 1H, t (7.7)	4.84, 1H, t (7.7)		
12´a	4.70, 1H, s	4.70, 1H, s	4.64, 1H, s	4.78, 1H, s	4.69, 1H, s	4.69, 1H, s	4.73, 1H, s	4.72, 1H, s		
12´b	4.65, 1H, s	4.65, 1H, s	4.57, 1H, s	4.72, 1H, s	4.64, 1H, s	4.65, 1H, s	4.69, 1H, s	4.69, 1H, s		
13´	1.75, 3H, s	1.74, 3H, s	1.69, 3H, s	1.78, 3H, s	1.69, 3H, s	1.74, 3H, s	1.76, 3H, s	1.75, 3H, s		
14´	1.25, 3H, d (6.9)	1.25, 3H, d (6.2)	1.12, 3H, d (6.9)	1.14, 3H, d (6.9)	1.20, 3H, d (6.8)	1.21, 3H, d (6.8)	1.08, 3H, d (7.3)	1.11, 3H, d (6.9)		
15´	1.63, 3H, s	1.65, 3H, d (1.3)	1.46, 3H, s	1.46, 3H, bs	1.89, 3H, d (1.5)	1.68, 3H, bs	1.62, 3H, s	1.63, 3H, s		

^{13}C NMR Spectroscopic Data (125 MHz, $CDCl_3$) for compounds **2-11**.

^{13}C	(**2**): δ_C, type	(**3**): δ_C, type	(**4**): δ_C, type	(**5**): δ_C, type	(**6**): δ_C, type	(**7**): δ_C, type	(**8**): δ_C, type	(**9**): δ_C, type	(**10**): δ_C, type	(**11**): δ_C, type
1	108.9, CH	108.8, CH	108.0, CH	108.8, CH	109.3, CH	111.54, CH	109.1, CH	107.9, CH	119.9, CH	120.1, CH
2	146.8, C	146.7, C	142.2, C	142.7, C	145.2, C	149.8, C	138.1, C	146.1, C	178.7, C	178.8, C
3	142.0, C	141.9, C	138.0, C	142.5, C	142.9, C	143.4, C	142.4, C	136.5, C	146.4, C	146.4, C
4	123.5, C	123.2, C	121.6, C	121.2, C	122.5, C	128.3, C	121.3, C	123.0, C	117.4, C	117.5, C
5	125.7, C	125.7, C	124.4, C	125.6, C	125.9, C	124.3, C	125.2, C	124.4, C	127.8, C	128.1, C
6	124.8, CH	124.5, CH	124.7, CH	124.7, CH	124.5, CH	187.9, C	124.5, CH	124.6, CH	134.3, CH	134.0, CH
7	128.9, CH	128.8, CH	128.6, CH	128.9, CH	128.9, CH	126.3, CH	128.8, CH	128.8, CH	118.5, CH	118.5, CH
8	45.1, CH	45.6, CH	45.8, CH	46.0, CH	44.7, CH	171.4, C	45.8, CH	45.9, CH	170.3, CH	169.0, CH
9	40.3, C	37.7, C	37.4, C	37.5, C	37.4, C	40.2, C	37.2, C	37.7, C	43.3, C	43.1, C
10	143.9, C	143.8, C	142.4, C	142.6, C	143.5, C	151.9, C	142.0, C	143.3, C	165.1, C	165.1, C
11	32.0, CH_2	31.2, CH_2	31.3, CH_2	31.5, CH_2	30.8, CH_2	34.3, CH_2	32.1, CH_2	31.4, CH_2	33.9, CH_2	34.1, CH_2
12	30.1, CH_2	30.2, CH_2	30.6, CH_2	30.3, CH_2	38.4, CH_2	28.7, CH_2	30.8, CH_2	30.8, CH_2	30.0, CH_2	30.3, CH_2
13	38.6, C	39.2, C	39.6, C	39.3, C	39.1, C	39.2, C	39.1, C	39.4, C	39.8, C	41.0, C
14	32.9, C	39.2, C	39.4, C	39.4, C	45.7, C	44.8, C	39.2, C	39.3, C	45.4, C	45.0, C
15	27.6, CH_2	28.4, CH_2	28.4, CH_2	28.5, CH_2	28.4, CH_2	30.0, CH_2	28.4, CH_2	28.5, CH_2	29.0, CH_2	28.9, CH_2
16	35.5, CH_2	35.7, CH_2	36.2, CH_2	36.8, CH_2	35.6, CH_2	35.6, CH_2	36.4, CH_2	36.8, CH_2	36.7, CH_2	35.9, CH_2
17	37.8, C	30.6, C	30.8, C	30.7, C	30.6, C	30.7, C	30.6, C	30.9, C	30.9, C	39.4, C
18	43.8, CH	44.6, CH	44.7, CH	44.2, CH	43.8, CH	44.5, CH	44.7, CH	44.9, CH	44.7, CH	43.9, CH
19	31.2, CH_2	30.8, CH_2	30.2, CH_2	30.4, CH_2	30.2, CH_2	31.0, CH_2	31.2, CH_2	31.3, CH_2	31.2, CH_2	32.4, CH_2
20	40.3, CH	40.8, C	40.7, C	40.9, C	40.7, C	40.6, C	40.7, C	40.9, C	40.8, C	42.3, CH
21	214.4, C	30.1, CH_2	30.2, CH_2	30.9, CH_2	30.1, CH_2	30.1, CH_2	29.6, CH_2	30.4, CH_2	30.2, CH_2	213.9, C
22	54.2, CH_2	36.7, CH_2	36.6, CH_2	37.3, CH_2	36.2, CH_2	36.6, CH_2	36.6, CH_2	36.4, CH_2	35.1, CH_2	52.9, CH_2
23	11.0, CH_3	11.0, CH_3	11.0, CH_3	11.1, CH_3	11.0, CH_3	13.3, CH_3	11.1, CH_3	11.4, CH_3	10.6, CH_3	10.6, CH_3
25	33.1, CH_3	22.4, CH_3	17.1, CH_3	22.7, CH_3	22.3, CH_3	37.9, CH_3	22.5, CH_3	22.6, CH_3	38.6 CH_3	38.5, CH_3
26	18.9, CH_3	17.2, CH_3	22.4, CH_3	17.2, CH_3	17.1, CH_3	21.0, CH_3	17.1, CH_3	17.2, CH_3	22.0 CH_3	21.9, CH_3

27	16.4, CH_3	17.7, CH_3	17.6, CH_3	17.8, CH_3	17.6, CH_3	18.5, CH_3	17.6, CH_3	17.8, CH_3	18.7 CH_3	20.1, CH_3
28	23.0, CH_3	32.0, CH_3	32.0, CH_3	32.2, CH_3	32.0, CH_3	31.8, CH_3	32.0, CH_3	32.2, CH_3	31.9 CH_3	32.9, CH_3
29		179.5, C	179.4, C	179.5, C	179.3, C	179.0, C	179.3, C	179.5, C	179.1 C	
30	15.3, CH_3	32.3, CH_3	32.3, CH_3	32.5, CH_3	32.3, CH_3	32.9, CH_3	32.3, CH_3	32.5, CH_3	33.0 CH_3	15.44, CH_3
OMe		51.8, CH_3	51.7, CH_3	51.9, CH_3	51.7, CH_3	51.7, CH_3	51.7, CH_3	51.9, CH_3	51.9 CH_3	
1′	93.0, C	92.9, C	87.4, C	91.5, C	92.5, C	92.9, C	140.2, C	135.8, C		
2′	38.9, CH_2	38.9, CH_2	42.0, CH_2	45.8, CH_2	36.6, CH_2	38.6, CH_2	36.2, CH_2	36.9, CH_2		
3′	128.3, CH	128.9, CH	124.3, CH	125.6, CH	128.5, CH	128.3, CH	74.5, CH	74.6, CH		
4′	140.7, C	140.7, C	151.4, C	142.3, C	140.9, C	140.8, C	90.1, C	90.2, C		
5′	97.4, C	97.3, C	84.6, C	90.6, C	97.0, C	97.0, C	134.7, C	138.1, C		
6′	38.3, CH_2	38.3, CH_2	35.4, CH_2	36.4, CH_2	39.2, CH_2	39.3, CH_2	30.2, CH_2	32.4, CH_2		
7′	44.1, CH	43.9, CH	45.5, CH	41.9, CH	45.7, CH	43.5, CH	49.6, CH	50.02, CH		
8′	35.7, CH_2	36.2, CH_2	39.1, CH_2	37.0, CH_2	33.0, CH_2	35.0, CH_2	30.2, CH_2	30.06, CH_2		
9′	27.6, CH_2	32.9, CH_2	39.2, CH_2	30.8, CH_2	31.4, CH_2	33.1, CH_2	40.1, CH_2	39.88, CH_2		
10′	42.4, CH	40.3, CH	33.1, CH	44.8, CH	39.7, CH	39.5, CH	42.8, CH	42.92, CH		
11′	151.23, C	151.2, C	152.1, C	151.8, C	151.4, C	151.1, C	150.9, C	151.05, C		
12′	108.7, CH_2	108.9, CH_2	108.6, CH_2	109.2, CH_2	108.8, CH_2	109.0, CH_2	108.8, CH_2	109.09, CH_2		
13′	20.0, CH_3	20.1, CH_3	19.6, CH_3	21.2, CH_3	20.1, CH_3	20.1, CH_3	20.7, CH_3	20.75, CH_3		
14′	18.9, CH_3	19.3, CH_3	20.5, CH_3	19.0, CH_3	19.3, CH_3	19.3, CH_3	17.3, CH_3	17.47, CH_3		
15′	12.7, CH_3	12.8, CH_3	23.0, CH_3	11.9, CH_3	12.7, CH_3	12.6, CH_3	14.1, CH_3	14.40, CH_3		

Table S1: ^{1}H and ^{13}C NMR data of known compounds (**2-11**).

VideoS1.avi

Video S1: Continuous live cell imaging of untreated SW1573 cells and cells exposed to **10** (1 μM, 20 h).
